# Lessons from Failed Attempts of Computationally Guided Synthesis of Aluminosilicate STF and IFR Zeolites in Hydroxide Media

**DOI:** 10.1021/acs.chemmater.5c01751

**Published:** 2025-12-09

**Authors:** Omer F. Altundal, Maria Galvez-Llompart, Angel Cantin, José Valero, Susana Valencia, Fernando Rey, Kingsley Christian Kemp, Suk Bong Hong, Frits Daeyaert, German Sastre

**Affiliations:** † Instituto de Tecnología Química UPV-CSIC, 83167 Universidad Politécnica de Valencia, Valencia 46022, Spain; ‡ Molecular Topology and Drug Design Unit. Faculty of Pharmacy and Food Sciences, Department of Preventive Medicine and Public Health, Food Sciences, Toxicology and Forensic Medicine, University of Valencia, Valencia 46100, Spain; § Center for Ordered Nanoporous Materials Synthesis, Division of Environmental Science and Engineering, 34995 POSTECH, Pohang 37673, South Korea; ∥ SynopsisDeNovoDesign, Beerse 2340, Belgium

## Abstract

Although zeolites are primarily aluminosilicates in terms of chemical composition, not all zeotypes have been successfully synthesized as aluminosilicates to date. This study aims to contribute answering the long-standing question of why some zeotypes cannot, or have not yet been, synthesized as aluminosilicates, using a computational approach that combines big data, artificial intelligence, accurate descriptors, and an algorithm for designing quaternary ammonium organic structure-directing agents (OSDAs) that help lower the total energy of the zeolite-OSDA system. The use of the synthesis energy descriptor enables evaluation of the reaction energy from gel monomers to the final zeolite product, including the occluded OSDA, while also accounting for the energetic contributions of framework aluminum, representing an improvement over previous models based on pure silica. The focus is on STF and IFR zeolites, whose synthesis in hydroxide media typically results in products with very high silicon to aluminum ratios. More than 10000 OSDAs, either designed or retrieved from existing databases, were evaluated under conditions likely to favor the formation of STF or IFR, and the corresponding synthesis energies were calculated for STF, IFR, and approximately 20 other competing zeolite phases. The computational results show that AEI, CHA, and a few other zeolites are consistently more energetically favorable, regardless of the OSDA used. This is explained by the increasing stability of phases as the aluminum content increases. Experimental synthesis using a shortlist of OSDAs predicted to favor STF or IFR, in addition to AEI or CHA, did not yield the expected aluminosilicate products, and instead, amorphous materials were obtained in most cases. While further refinement of computational tools is needed to better reflect experimental synthesis conditions, the results provide valuable insight and justification for the difficulty of synthesizing aluminosilicate STF and IFR in hydroxide media.

## Graphical abstract

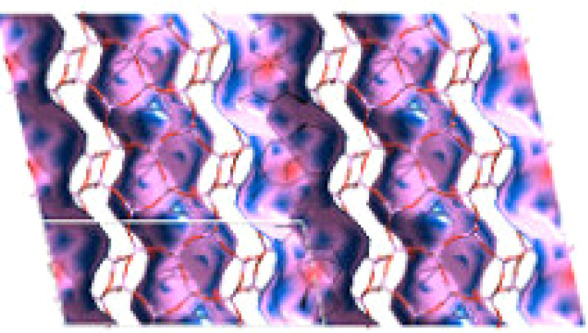

## 1 Introduction

Zeolites are one of the most influential materials in the chemical industry due to their unique chemical and structural properties, making them ideal candidates for gas separation, ion exchange, and catalysis. Zeolites were first discovered and applied in industry as aluminosilicates,^1,2^ which continues to be one of the most important chemical composition targets for zeolites. For acid catalysis, one of the main applications of zeolites, a compromise between the number of Brønsted acid sites (lower Si/Al ratio, leading to more sites) and strength (larger Si/Al ratio, leading to stronger sites) is desired.^3^ In fact, considerable research has been devoted to controlling the number and strength of acid sites and their location for decades.^4–16^

Organic structure-directing agents (OSDAs) play an essential role in the phase determination of zeolite synthesis due to their short-range interactions with the zeolite micropores.^17–19^ However, some other factors influence the synthesis outcome, such as the presence of fluoride^20–26^ and aluminum^27–30^ in the synthesis gel. Fluorides are extra-framework anions located inside the small pores of the zeolite, whereas aluminum replaces framework silicon atoms to generate [AlO_4/2_]^−^ tetrahedra, and these negative charges are usually compensated by the cationic nature of most OSDAs. The match in size and shape between the OSDA and the zeolite micropores contributes to the strengthening of the short-range van der Waals stabilization which is one of the driving energetic terms partially controlling the zeolite phase outcome. This van der Waals zeolite-OSDA interaction plays a more prominent driving role in pure silica zeolites, while in the case of aluminosilicate zeolites the electrostatic [AlO_4/2_]^−^-OSDA^+^ interactions also contribute significantly to drive the synthesis toward specific zeolite phases.^31^ In addition to framework Al, defects such as silanol nests may help compensate OSDA charge, especially in hydroxide media. These defects, that have been taken into account in a previous study,^32^ can shift the synthesis outcome due to changes in the framework energetics and the local charge balance around the OSDA. In this study, however, we did not explicitly consider defects in the computational analysis and focused only on framework Al as the source of charge compensation. Early computational studies were aimed to clarify how dominant OSDAs are to drive the synthesis of pure silica zeolites.^33–36^ With the improvement of computational technologies and development of zeolite and OSDA databases, recent studies about pure silica zeolites have started making use of artificial intelligence and machine learning methods.^37–39^ Schwalbe-Koda et al.^40^ calculated the binding affinity of 549 OSDA structures and 209 zeolites and created the Organic Structure-directing agent DataBase (OSDB).^41^ They also used energetic and physical descriptors in this database to propose an OSDA (tris­(dimethylamino)­(methyl)­phosphonium) for AEI, which successfully directed the synthesis of the desired zeolite phase. Daeyaert and Deem utilized their de novo algorithm to design putative OSDAs for zeolite *BEA.^42^ They found that among the hundreds of thousands of molecules they designed, 212 of them had stabilization energies lower than −15.0 kJ (mol Si)^−1^, which is significantly lower than the lowest stabilization energy of the OSDAs used in the synthesis of *BEA (−14.1 kJ (mol Si)^−1^), while having at least +2.0 kJ (mol Si)^−1^ higher stabilization energies in BEB. Aluminosilicate zeolites have also been included in recent computational studies.^43–46^ Rojas et al.^47^ combined experimental and computational methods to investigate the structure direction of 1-benzyl-3-methylimidazolium (1B3MI) and 1-benzyl-2,3-dimethylimidazolium (1B23DMI) cations. Both OSDAs can synthesize MTW at high H_2_O/Si ratios, while only 1B3MI can synthesize MFI at lower H_2_O/Si ratios, which is explained by the low stabilization energy of 1B23DMI cations inside the MFI 10-ring channels calculated by molecular mechanics (−76.4 kcal mol^–1^ for 1B23DMI and – 86.2 kcal mol^–1^ for 1B3MI). Oishi et al.^48^ employed computational techniques to investigate the aluminum-directing effect of various OSDAs in FER and CHA. They concluded that the aluminum-directing effect is stronger for OSDAs with N–H moieties, i.e., tertiary ammonium cations, than quaternary ammonium cations due to the strong interaction between O­(−Al) and N–H groups.

Taking all factors that energetically influence the zeolite phase outcome into account, we have recently put forward an equation that includes a descriptor called synthesis energy (*E*_syn_). This equation is valid for pure silica and aluminosilicate compositions, with and without fluoride present, and so far has been able to predict the zeolites obtained in aluminosilicate and silicate synthesis gels when using specific OSDAs that give different zeolites in either of these chemical compositions.^49,50^ These studies considered a small number of competing zeolites obtained from synthesis experiments for each OSDA. Employing molecular simulations, we determined the stabilization energy of each competing zeolite-OSDA pair and this was then used to calculate the *E*_syn_’s of zeo-OSDA systems. For each OSDA, considering all competing zeolites, the lowest *E*_syn_ of zeo-OSDA pairs gives the computational prediction of the zeolite phase product. Notably, we could correlate the aluminosilicate phase formed with that for which the Si/Al ratio is lowest; hence, the larger the Al content, the larger the stability of the phase formed (Table S1). For a given OSDA and a given aluminosilicate starting gel, the competing phase of lowest energy will be the one able to accommodate more OSDA cations per tetrahedral atom (T atom) since this leads to a larger Al content or lower Si/Al ratio and hence a lower energy. This correlation between aluminum content and energetic stabilization has also been consistently reported in previous studies. Thermochemical measurements showed that the enthalpy of formation becomes increasingly exothermic with higher framework Al substitution, reflecting stronger Coulombic interactions between the framework and the charge-compensating cations.^51^ Periodic DFT calculations further confirmed that the substitution of silicon by aluminum, together with charge-compensating cations, decreases the lattice energy and reduces framework strain, which makes structures with higher Al content more stable.^52^ In agreement, synthesis experiments in purely inorganic media revealed that zeolite topologies capable of hosting a larger number of cations per framework unit and therefore containing more aluminum are preferentially stabilized during crystallization.^53^ More recently, phase selection studies have shown that the way cations exchange and interact with the framework plays a key role in determining which zeolite structure forms and how stable it becomes.^54^

In the present study, we aim to apply the predicting value of this computational tool to the synthesis of the aluminosilicate version of STF and IFR (AlSi-STF and AlSi-IFR, respectively) as our target zeolites. STF has not yet been obtained as an aluminosilicate, and although IFR has been synthesized as an aluminosilicate, it typically requires environmentally unfriendly fluoride-based media and expensive OSDAs. Hence, for our targets we will also impose the condition to the synthesis to be carried out in hydroxide media. STF is a one-dimensional medium pore zeolite, with 10-ring straight channels of approximate 5.4 × 5.7 Å, which shows great potential as a catalyst in *n*-alkane isomerization processes, such as the case of ZSM-22 (TON), also with monodimensional medium pore.^55,56^ IFR is a large-pore zeolite with a monodimensional channel system consisting of undulating 12-ring channels of ca. 6.2 × 7.2 Å. Its framework contains the small *bea* ([4^3^5^2^6]) cavity, in which fluoride anions are located, which facilitates the synthesis under aluminosilicate chemical composition. Both frameworks are illustrated in Figure 1.

**Figure 1:**
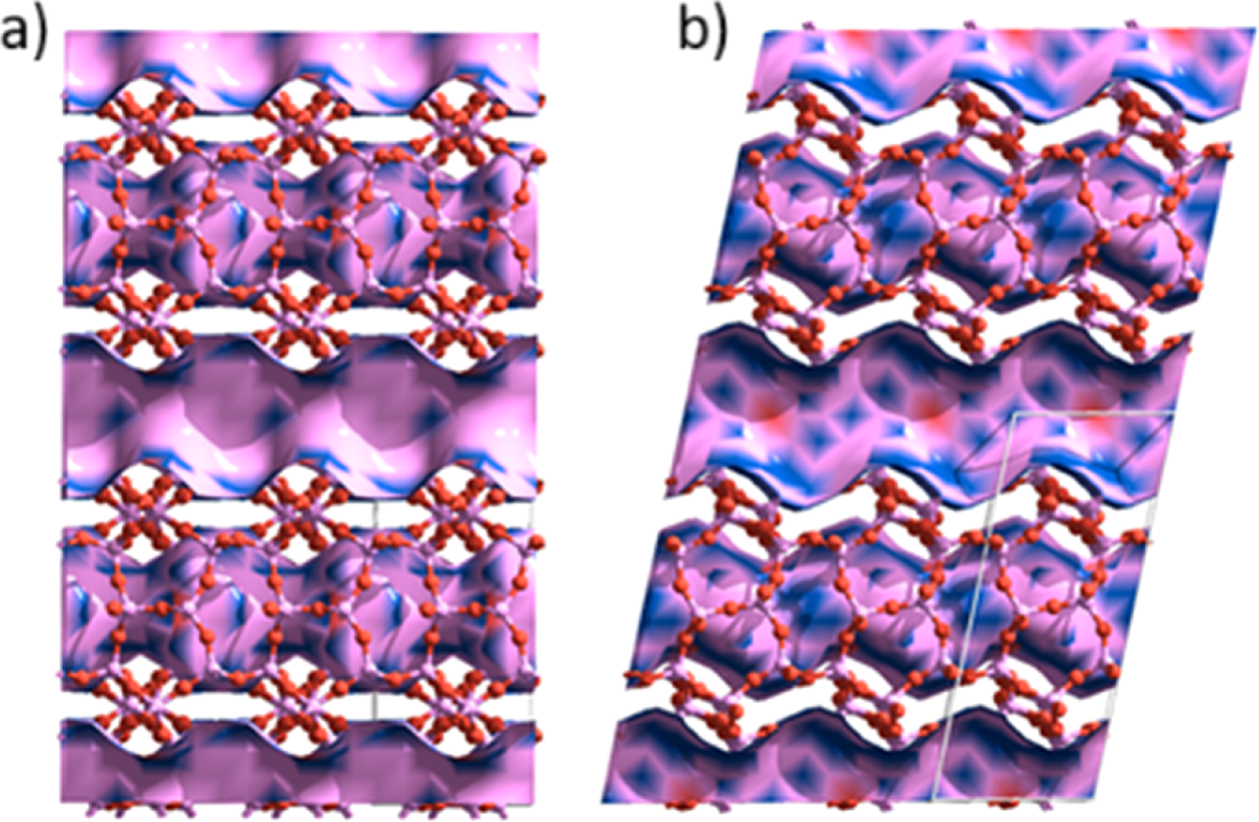
Unit cell representations of a) STF and b) IFR.

Many OSDAs were reported in the literature to synthesize silica STF (Si-STF; 36 OSDAs reported in OSDB^40^), which means we have abundant information on the characteristics that a good OSDA for STF should fulfill from the point of view of zeo-OSDA van der Waals interactions. IFR, on the other hand, has only been synthesized as aluminosilicate using expensive benzylquinuclidinium cations.^12,57^ Thus, finding a novel and more accessible OSDA for the synthesis of aluminosilicate IFR in hydroxide media is an interesting target. Through these efforts, we seek to advance the rational design of OSDAs, making zeolite synthesis more efficient, cost-effective, and useful for catalytic applications.

## 2 Computational Methods

### 2.1 Determination of Competing Zeolite Phases

To identify the competing zeolite phases for the target zeolites, we employed two strategies based on the availability (“High” or “Low”) of OSDAs used for their synthesis (Figure 2).

**Figure 2:**
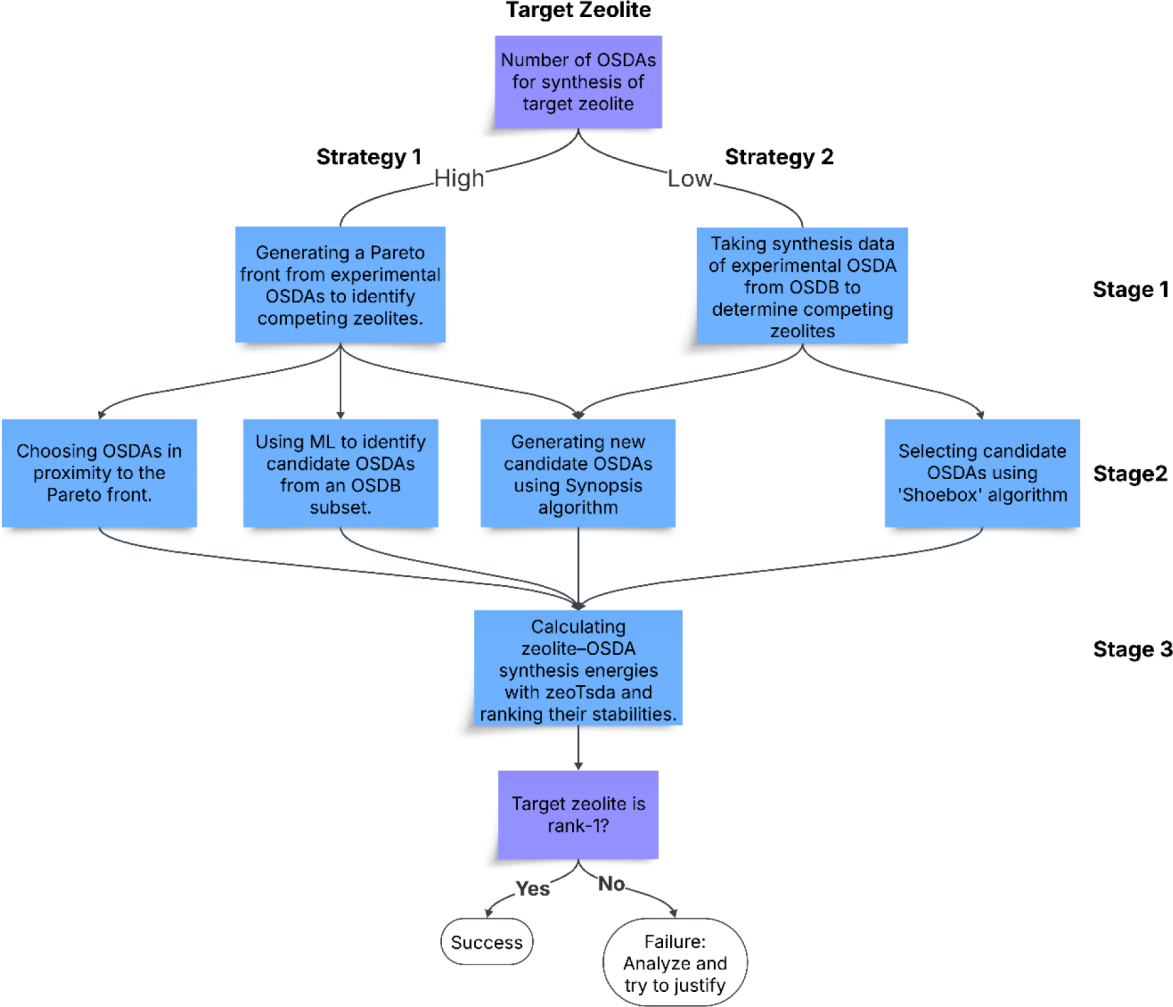
Two computational strategies used in this study in order to suggest candidate OSDAs for the synthesis of target aluminosilicate zeolites. For STF, Strategy-1 will be followed (Figure 5), and for IFR, Strategy-2 will be followed (Figure 6).

**Strategy-1** . High-silica STF is reported to have been synthesized with 36 OSDAs in OSDB.^58^ For these known, “active”, OSDAs, we calculated the stabilization energies in pure silica STF using the zeodock algorithm (see Section 2.4). We performed the same calculation for all OSDAs reported in OSDB to generate alternative zeolites, which we term “inactives”. In Figure 3, we plotted the calculated stabilization energies in STF of these “active” (green dots) and “inactive” (red dots) OSDAs against their molecular volume, and performed a linear regression to the set of actives. This regression line (black line in Figure 3) coincides with the Pareto front and represents the optimum balance between stabilization energy and molecular volume for successful OSDAs for STF. Competing zeolite phases were identified as those, non-STF, phases formed by the OSDAs close to this Pareto front.

**Figure 3:**
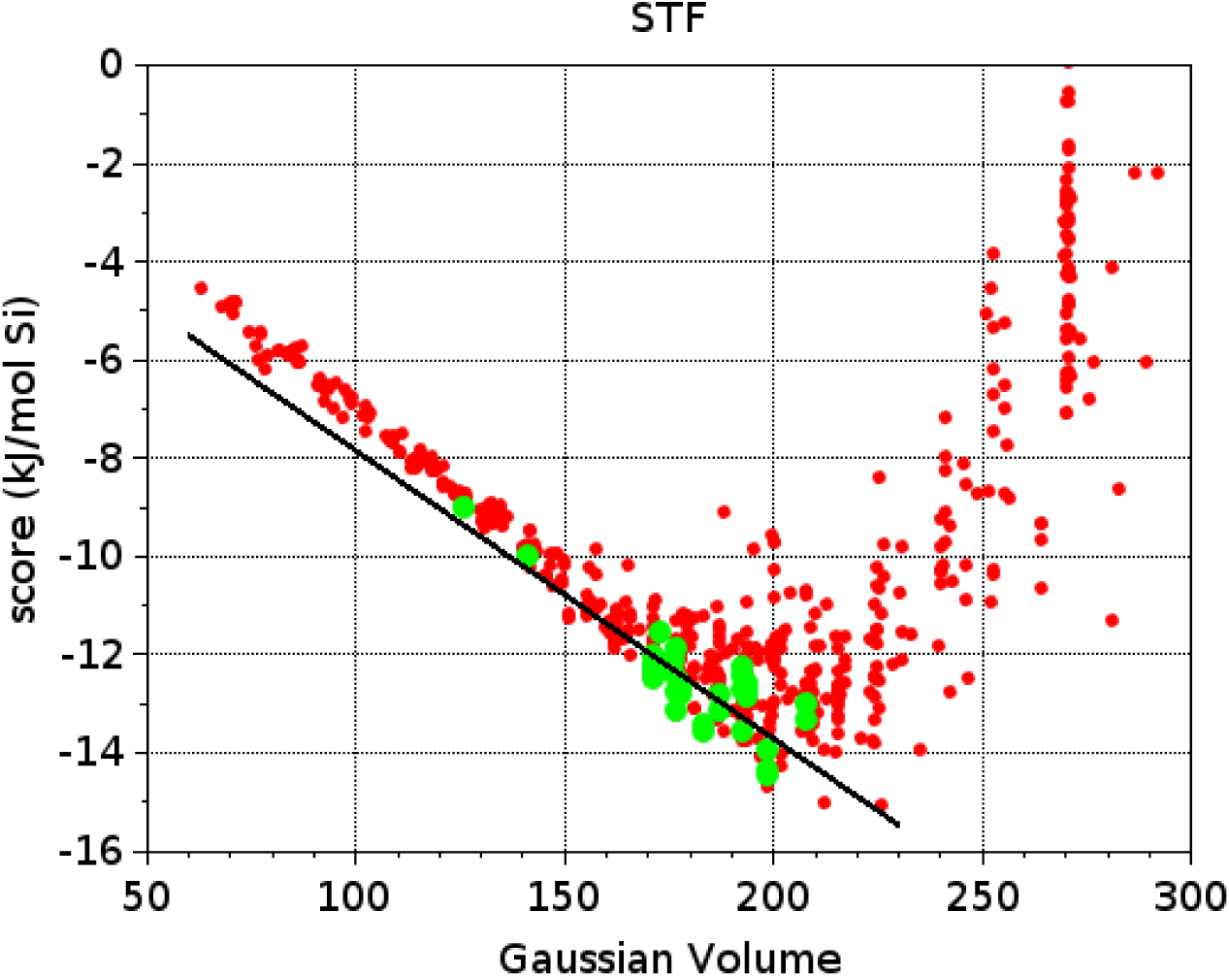
Stabilization energy (score) in STF vs Gaussian volume (Å^3^) of OSDAs in OSDB. Green dots correspond to OSDAs reported in the literature for the synthesis of high silica STF. Red dots correspond to OSDAs reported in the OSDB to form alternative zeolite phases. The black line corresponds to the Pareto front for the active OSDAs for STF.

To identify putative OSDAs for AlSi-STF we followed four approaches: (i) Initially, we selected OSDAs that are used to synthesize the pure silica target zeolite, as they are strong candidates for synthesizing its aluminosilicate version. (ii) From Figure 3, we selected OSDAs not reported to template all-silica STF but close to the Pareto front, as these exhibit the optimal molecular volume. (iii) From OSDB, we selected OSDA structures for which no zeolite synthesis results have been reported and performed virtual screening using machine learning and AI models trained on reported zeolite syntheses in this database (Section 2.2); and (iv) Finally, we used de novo design methods to generate putative OSDAs (Section 2.3).

**Strategy-2** . High-silica IFR has been synthesized with only a few OSDAs, and therefore the available statistics are insufficient to construct a Pareto front from the plot of energy vs volume. Therefore, we identified competing zeolite phases as those zeolites obtained using the OSDAs that also produced high-silica IFR. To initially identify putative OSDAs for AlSi-IFR, we employ two approaches: (i) we extracted the “Shoebox”^59^ dimensions (XYZ) of OSDAs, defined as the length, width, and height of the smallest axis-aligned box containing the optimized cation, that successfully synthesized the all-silica target zeolite and used these parameters to search for structurally similar OSDAs in OSDB database; and (ii) Similarly to Strategy-1, we generated OSDAs using de novo design methods to expand the pool of potential candidates.

With the initial OSDA lists obtained from both strategies and having identified the competing zeolites for both target zeolites, we proceeded with the established workflow: calculating the *E*_syn_’s of these OSDAs with the target zeolites using zeoTsda (Section 2.5) and evaluating their relative stabilities, considering an aluminosilicate composition, in order to assess their potential for zeolite synthesis.

### 2.2 Training of Machine Learning and Artificial Intelligence Models: STF-Templating OSDAs

To further explore whether there are promising candidates among the unpublished OSDAs in OSDB (subset that we call “OSDAs not in papers”), we trained machine learning (ML) and artificial intelligence (AI) models using the dataset of literature-reported OSDAs known to direct STF synthesis. These models were then applied to screen the unreported OSDA subset and identify potential candidates for experimental evaluation.

Both ML and AI models were implemented using StatSoft^60^ through linear discriminant analysis (LDA) and artificial neural networks (ANN). Different training strategies were employed within the three models. (a) LDA Model #1:25% of all available data was used for external validation of the model (external test set), while the remaining data was used to train the LDA model. (b) ANN Model #2:25% of the data was allocated to the external test set for model external validation. From the remaining, 80% was assigned for training the ANN, and the remaining 20% was set aside for internal validation (validation set). (c) ANN Model #3: This model was trained using all available OSDAs known to direct STF synthesis, while the inactive category comprised OSDAs that do not lead to STF zeolite formation and share chemical similarity with the active OSDAs. This model was trained using 80% of the available data and internally validated with the remaining 20% (validation set). Since all active OSDAs leading to STF zeolite formation were employed for training and internal validation of the model, the external test set consisted only of the remaining inactive compounds that were not used in training ANN Model #3 and did not share chemical similarity with active OSDAs.

### 2.3 Generation of Quaternary Ammonium OSDAs: de Novo Design, Stochastic Virtual Combinatorial Chemistry, and Stochastic Multiple Virtual Combinatorial Chemistry

Computational design of OSDAs for zeolites with de novo design was pioneered in the seminal 1996 paper by Lewis et al.^61^ In recent years, de novo design,^62^ stochastic^63^ and exhaustive virtual combinatorial chemistry,^64^ and generative neural network algorithms^65^ have been developed for automated design of OSDAs for zeolites. In the present work, we used the Synopsis de novo design algorithm originally developed in drug design and adapted to and extensively applied to the design of OSDAs.^66^

This algorithm generates synthesis routes to molecules to ensure their synthetic accessibility, and uses an independent, user-provided multiobjective scoring function to calculate the molecular properties that need to be optimized. For many zeolite frameworks, charged OSDAs containing a quaternary ammonium functionality have been found to be the most successful templating agents. The presence of this functionality in the de novo designed OSDAs has been enforced by introducing it as a filter in the scoring function of the de novo design algorithm.

A more direct way to ensure the presence of quaternary ammonium in designed OSDAs is by virtual combinatorial chemistry. Hereby, a fixed reaction scheme leading to a quaternary ammonium end product is chosen. The reaction scheme can consist of a single reaction or a reaction sequence. The reaction scheme and a set of available starting materials define a chemical search space consisting of all the end products that can be synthesized using all combinations of the starting materials. For reaction schemes consisting of multiple reactions and for which many starting materials are available, this search space becomes too large for an exhaustive evaluation or even enumeration. Therefore, a stochastic optimization algorithm is typically applied to search for the set of starting materials that generates high scoring molecules for the given reaction scheme. Documented examples of this approach use a genetic algorithm,^67^ or an ant-colonization algorithm.^63^ The reaction scheme used in this work to design quaternary ammonium OSDAs directed toward STF is shown in Figure S1. Details of the stochastic virtual combinatorial chemistry and de novo design algorithms are described in the Supporting Information (Section S3).

While the use of a fixed reaction scheme in stochastic virtual combinatorial chemistry ensures that the designed end products contain the required functionality, the search space covered, even when large, is limited by considering one single synthesis scheme. We have therefore devised a stochastic multiple combinatorial chemistry algorithm that simultaneously searches multiple reaction schemes. For this purpose, a number of reaction schemes are organized in a tree structure as shown in Figure 4. The top of the tree corresponds to the end product, and the leaves of the tree correspond to the starting materials. The nodes of the trees are connected by edges that correspond to well-documented organic chemistry reactions. A virtual reaction route is defined by a path starting at one or several leaves leading toward the top of the tree through the available edges. The setup of the tree is chosen such that all possible paths lead to reaction products that contain the desired quaternary ammonium chemical functionality. A genetic algorithm is used to find the combination of reaction routes and starting materials within the tree structure that maximize the value of a scoring function as applied to the end product of the synthesis route. As with the combinatorial chemistry and de novo design algorithms, the scoring function is multiobjective and independent of the molecule generation. The scoring function used in this work is summarized in Table S3, which calculates the stabilization energy of the designed molecules in a target zeolite, while applying filters to control the flexibility, size and chemical stability under zeolite synthesis conditions. Appropriate reagents to participate in the individual reactions with a listed price of no more than 100 USD/gram were selected from ChemSpace.^68^ Based upon the number of reagents, and not taking into account identical molecules that can be generated by multiple routes in the synthesis tree, the chemical search space was estimated to contain 6.5•10^10^ molecules. This space is searched by a genetic algorithm: a population of 100 random synthesis trees is generated and then evolved by applying selection, crossover and mutation operators. The selection operator is a tournament selection applied to the Pareto-sorted population.^62^

**Figure 4:**
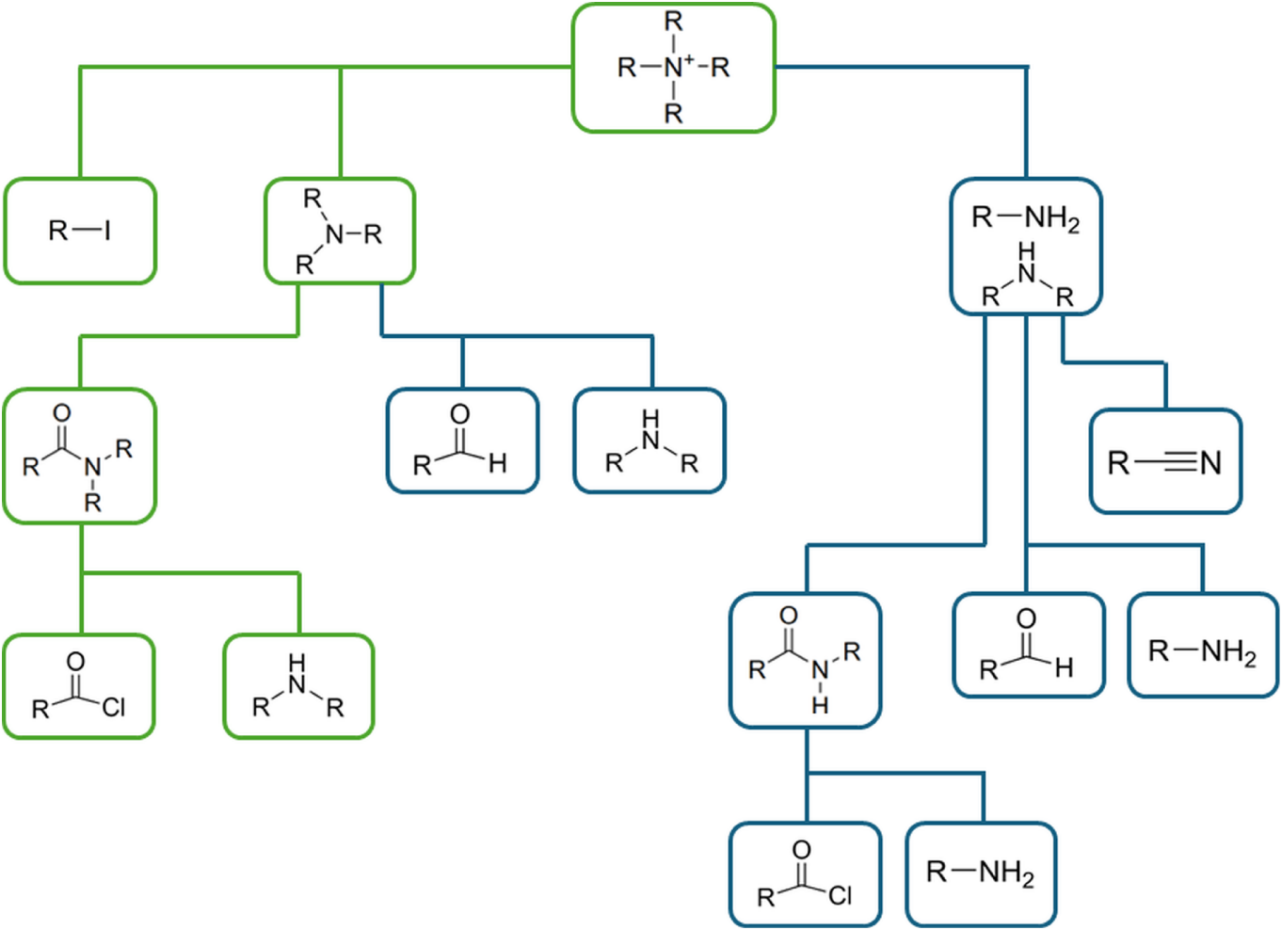
Multiple combinatorial chemistry tree used to generate quaternary ammonium cations in this work. Nodes and edges in green correspond to the reaction sequence in Figure S1.

### 2.4 The Zeodock Algorithm to Calculate Zeolite-OSDA van der Waals Interaction

The zeodock algorithm has been developed to efficiently and reproducibly estimate the short-range van der Waals stabilization energy of an OSDA in a pure silica zeolite framework. The algorithm is composed of four stages: random placement of single OSDA copies into the zeolite unit cell, selection of tuples of OSDA copies that do not overlap with one another, rigid optimization of the van der Waals energies of the selected tuples in rotation-translation space, and frozen pose molecular mechanics energy minimization of the OSDA-zeolite complexes. In contrast to the zeoTsda software suite, zeodock only considers the van der Waals contribution to zeolite-OSDA interaction in pure silica frameworks and therefore does not consider charged species. Due to its computational efficiency, however, it is well suited for initial rapid screening of large numbers of OSDAs. In this study, it was used in the de novo design and stochastic combinatorial chemistry algorithms to evaluate the zeolite-OSDA interactions. Details of the zeodock algorithm are given in the Supporting Information (Section S2).

### 2.5 *E*_syn_ Calculations

To evaluate the stabilities of competing zeolite phases, we employed our *E*_syn_ descriptor (synthesis energy), which accounts for the energies of all components in the synthesis equation, similarly to an enthalpy of reaction. Our previous studies have demonstrated the effectiveness of this method in accurately predicting the phase selectivity of aluminosilicate zeolites.^49,50^

Synthesis reactions to obtain two zeolites from the same initial gel are as follows:

1 $$p_{1}\cdot \mathrm{OSDAOH}+m_{1}\cdot {\mathrm{Si}(\mathrm{OH})}_{4}+p_{1}\cdot {\mathrm{Al}(\mathrm{OH})}_{3}\to {\mathrm{zeo}}_{1}-\mathrm{OSDA}\left(m_{1}{,p}_{1}\right)+w_{1}\cdot H_{2}O$$

2 $$p_{1}\cdot \mathrm{OSDAOH}+m_{1}\cdot {\mathrm{Si}(\mathrm{OH})}_{4}+p_{1}\cdot {\mathrm{Al}(\mathrm{OH})}_{3}\to {\mathrm{zeo}}_{2}-\mathrm{OSDA}\left(m_{2},p_{2}\right)+w_{2}\cdot H_{2}O+m_{3}\cdot {\mathrm{Si}(\mathrm{OH})}_{4}+p_{3}\cdot {\mathrm{Al}(\mathrm{OH})}_{3}$$

Eqs 1 and 2 represent the synthesis equations for different aluminosilicate zeolite phases starting from the same reactants. A zeolite (zeo_1_) with a Si/Al ratio of *m*_1_/*p*_1_ was synthesized, generating water as a byproduct. In contrast, another zeolite (zeo_2_) with a Si/Al ratio of *m*_2_/*p*_2_ was produced with a different quantity of water consequently, along with an excess of Si and Al monomers (*m*_3_ and *p*_3_, respectively). This experimental design allows for a direct comparison of the energetic stability of the resulting products, as both reactions originate from the same set of reactants. A comprehensive analysis of the energies of both reactions leads to the derivation of an equation that quantitatively assesses the stability of zeolite-SDA pairs based on their synthesis processes.

3 $$E_{\mathrm{syn}}=E_{\mathrm{zeo}-\mathrm{OSDA}}+2\times E_{H_{2}O}-\frac{p}{m+p}\times \left(E_{\mathrm{OSDAOH}}+E_{{\mathrm{Al}(\mathrm{OH})}_{3}}\right)-\frac{m}{m+p}\left(E_{{\mathrm{Si}(\mathrm{OH})}_{4}}\right)$$

Here, *E*_syn_ denotes the synthesis energy of the reaction, while *E*_zeo‑OSDA_ represents the energy of a zeolite-OSDA pair with a specific Si/Al ratio. The term *E*_OSDAOH_ corresponds to the energy of OSDA neutralized by hydroxide anions. The number of aluminum atoms is given by *p*, and *m* accounts for the silicon atoms, leading to *m+p* T atoms. Furthermore, to ensure the electroneutrality condition is fulfilled, the number of Al atoms (*p*) is counterbalanced by the number of OSDAs (*p*) resulting in net zero charge balance. Comparison between the stabilities of aluminosilicate zeolite phases for each OSDA was done based on the *E*_syn_’s by selecting the structure with the lowest *E*_syn_ as the most stable structure. These energies were calculated by force field simulations implemented in zeoTsda software,^69^ using XYZ (for OSDA) and CIF (for zeolite) files as the only input, which calculates optimum OSDA loading using MC (Monte Carlo) and LEM (lattice energy minimization) techniques. The calculation, with the help of zeoTAl software, is performed on a large (user defined) number of randomly generated Al distributions, of which those resulting with the lowest energies are selected for the final calculation of *E*_syn_. The procedure is automatically repeated for a double entry (big data) list of OSDAs and zeolites. More details are given in Supporting Information (Section S4).

## 3 Synthesis of OSDAs and Zeolites

The final list of candidate OSDAs resulting from the procedures described in Figures 4 (for STF) and 5 (for IFR) are summarized in Section S7, Tables S14–S17. These are the most promising OSDA candidates for the synthesis of AlSi-STF and AlSi-IFR. From these, 3 OSDAs were selected and synthesized. Zeolite synthesis was performed in the presence of these OSDAs, by varying and adjusting the synthesis conditions in hydroxide media expected to be more favorable to obtain the target aluminosilicate zeolites. A concise summary of the hydrothermal conditions evaluated is given in Table 1. The final results are discussed in Section 4.4. Full details of the synthesis of OSDAs and zeolites are described in Supporting Information (Sections S8 and S9).

**Table 1:** Summary of Hydrothermal Conditions Evaluated for STF and IFR Syntheses in Hydroxide Media

Target	Gel composition	Si/Al range	Inorganic cation	*T* (°C)	Time (days)
STF	SiO_2_: *x* Al_2_O_3_: 0.5 R(OH): 10 H_2_O	∞–50	none	150–200	14–18
IFR	SiO_2_: *x* Al_2_O_3_: 0.5 R(OH): 10 H_2_O	∞–50	none	175–200	14–28
IFR, Na-present (SSZ-42-type)	SiO_2_: *x* Al_2_O_3_: *y* Na_2_O: *z* R(OH): 43.3 H_2_O	10–20	Na^+^ (0–0.18)	150–165	14
IFR, K-present (MCM-58-type)	SiO_2_: *w* Al_2_O_3_: *x* K_2_O: *y* R(OH): *z* RI: 40 H_2_O	10–100	K^+^ (0–0.29)	175	7

**Figure 5:**
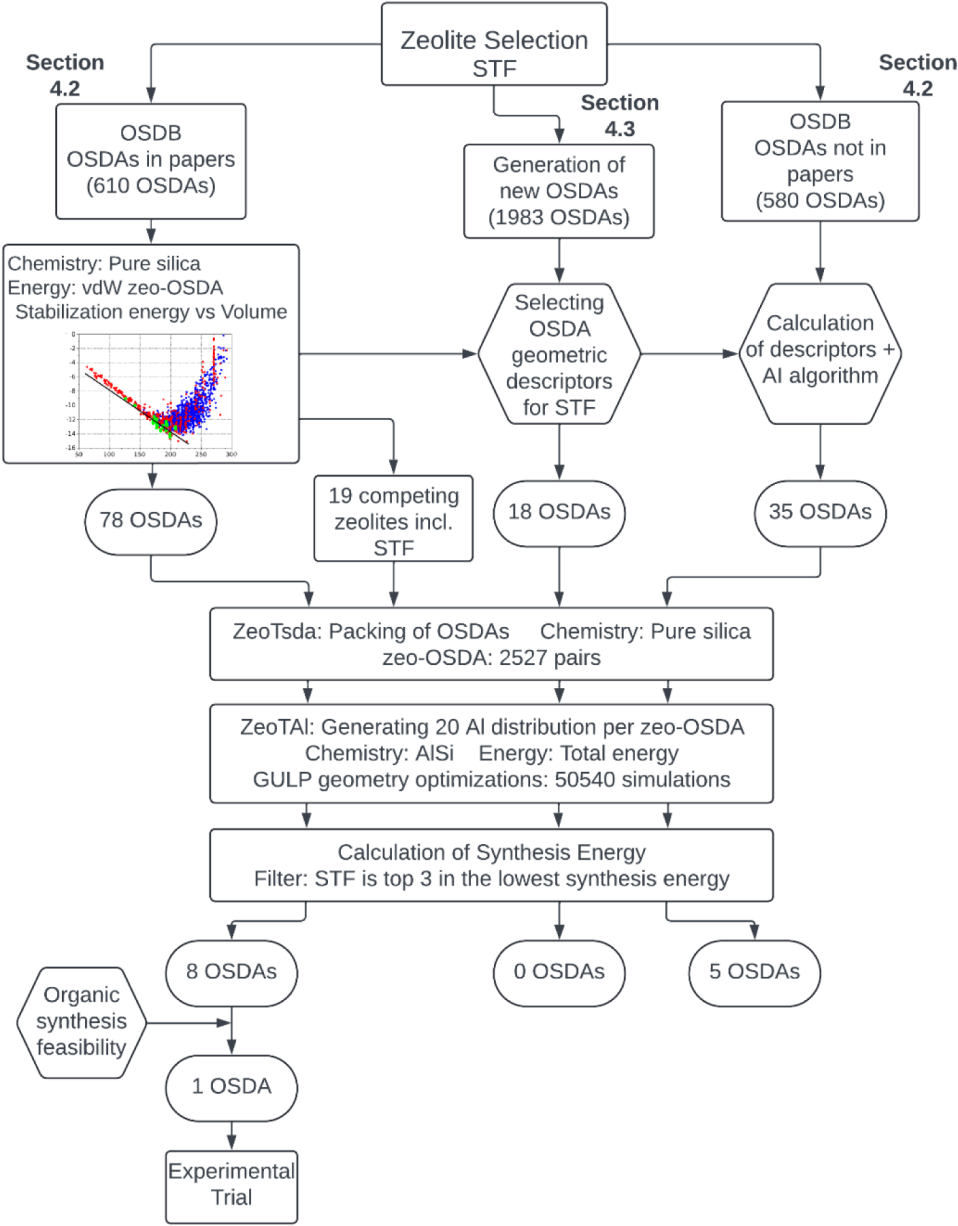
Computational “Strategy-1” (see Figure 2) used in this study for designing OSDAs for targeted AlSi-STF synthesis.

## 4 Results and Discussion

### 4.1 Selection of Competing Zeolite Phases for STF and IFR

The computational methods employed in this study are summarized in Figure 2, which outlines two strategies to determine competing zeolite phases.

#### 4.1.1 Using Strategy 1: Identification of Competing Phases for STF

We utilized OSDB^58^ to identify competing zeolite phases for our target zeolite, STF. The workflow for determining competing phases and potential OSDAs for STF is depicted in Figure 5.

In the first step, we calculated the van der Waals stabilization energies for 610 OSDAs from the updated OSDB that correspond to published synthesis data. These energies were computed using zeodock^42^ and plotted against OSDA molecular volume (Figure 3). We classified these OSDAs into two categories: Active OSDAs, which successfully direct the synthesis of high-silica STF (marked as green points in Figure 3), and inactive OSDAs which do not facilitate STF synthesis.

We then observed that all active OSDAs were clustered close to a straight-line fit in the stabilization energy vs volume plot. This line defines the Pareto front, representing the optimal balance between stabilization energy and molecular volume for synthesis of STF. After analyzing the points, we identified 42 OSDAs that were close to the linear fit (∓ 0.2 kJ/mol Si) but were classified as inactive. Although these OSDAs exhibit favorable stabilization energies for STF, they direct the synthesis of high-silica zeolites other than STF. The 18 pure silica zeolites that are synthesized with these “inactive-close” OSDAs were identified as potential competitors for STF: AEI, *BEA, BEC, CHA, DDR, DOH, EUO, IFR, ITE, LTA, MEL, MFI, MOR, MTW, NON, RTH, SFF, SGT. For AlSi-STF, additional energetic factors beyond van der Waals stabilization energy become important. Since all “inactive-close” OSDAs exhibit favorable stabilization energy within the pure silica STF (Si-STF) framework, they are potential candidates for AlSi-STF synthesis, competing against the 18 zeolite frameworks listed above. Additionally, the active OSDAs, which already facilitate high-silica STF synthesis, are also strong candidates for AlSi-STF, as their ability to stabilize the all-silica framework suggests they may successfully promote the aluminosilicate form.

To determine the most promising OSDAs for AlSi-STF, we employed aluminosilicate *E*_syn_ calculations, to evaluate all OSDA-zeolite pairs (including STF and the 18 competing zeolites). This methodology provided a reliable prediction of which OSDAs minimize *E*_syn_ for AlSi-STF. Details are included in Supporting Information (Section S7). The complete selection process for STF candidates is summarized in Figure 5.

#### 4.1.2 Using Strategy 2: Identification of Competing Phases for IFR

The identification of competing zeolite phases for IFR followed a different approach due to the limited number of known OSDAs known for IFR synthesis, as depicted in Figure 6. Unlike STF, where a Pareto front could be constructed, the lack of a large number of OSDAs to synthesize IFR required an alternative strategy. Since IFR was already identified as a competing phase for STF, we included all STF competing phases as competitors for IFR as well. Additionally, we examined other zeolites synthesized using the active OSDAs from OSDB that were linked to IFR synthesis. This analysis identified three additional competing zeolite phases: AFI, CON, and *STO. Thus, the final list of competing phases for IFR included all 19 competing STF phases, plus AFI, CON, and *STO.

**Figure 6:**
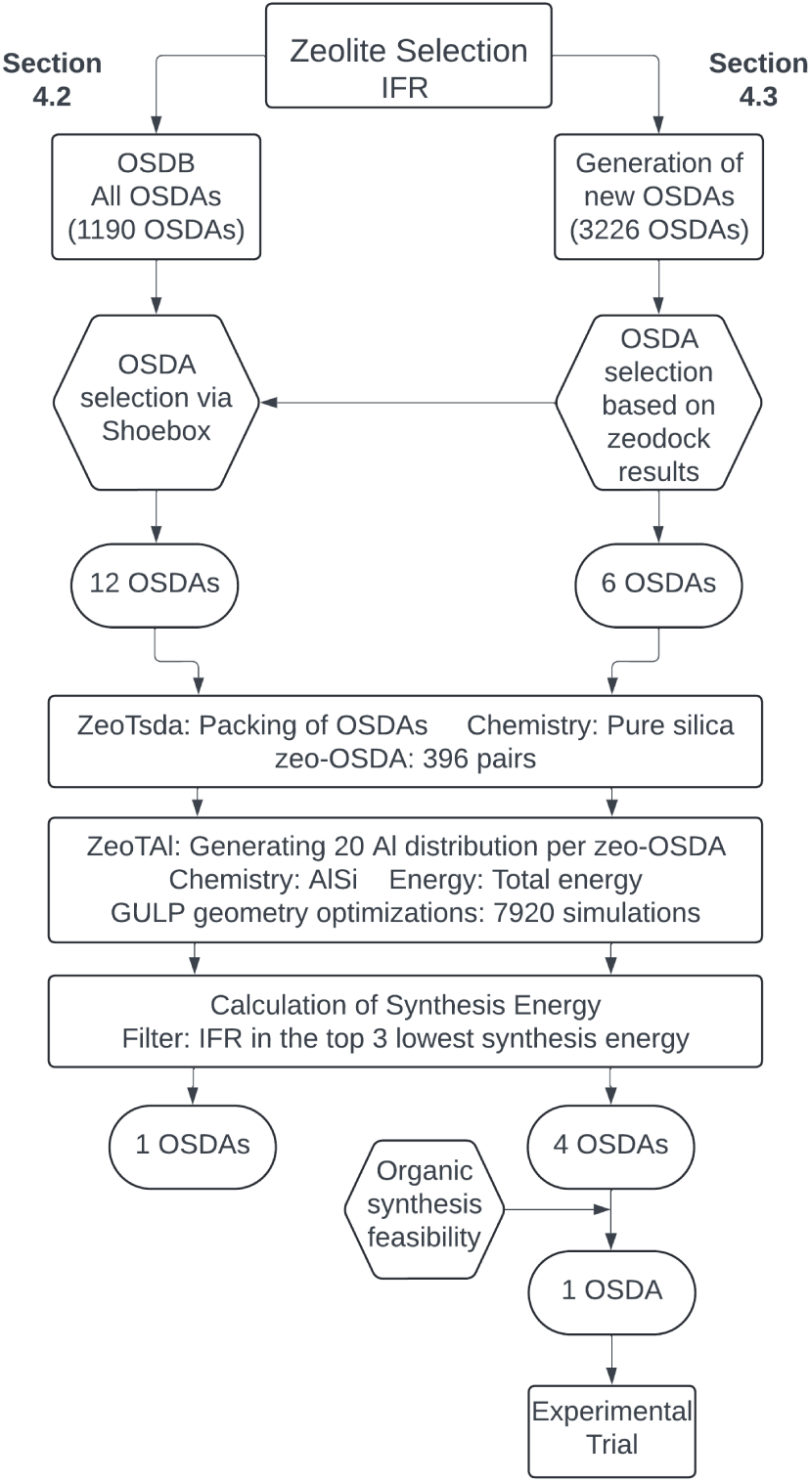
Computational “Strategy-2” (see Figure 2) used in this study for designing OSDAs for targeted AlSi-IFR synthesis.

With the competing zeolite phases identified for both STF and IFR, we proceeded to assess the stability of these zeolites when paired with candidate OSDAs. The complete selection process for IFR candidates is summarized in Figure 6.

### 4.2 Search of OSDAs from OSDB

#### 4.2.1 Search of OSDAs from OSDB for STF

OSDB contains 1190 OSDAs divided in two categories, that here are called “OSDAs in papers” and “OSDAs not in papers”. The first one consists of 610 OSDAs previously used in published studies to synthesize zeolites, with their synthesis data available in OSDB. The second set contains 580 molecules not yet described in the literature as OSDAs. The search methods for these two subsets are described separately.

##### 4.2.1.1 OSDAs in Papers

After selecting the competing phases, we proceeded with the analysis of a subset within OSDB, known as “OSDAs in papers”. From this subset, we focused on the 42 “inactive” OSDAs that are close to the Pareto front in Figure 3, hence referred to as “inactive-close”, and on the 36 known active OSDAs for STF. The InChIKeys and SMILES representations of these OSDAs are included as Supporting Information (Table S6). To assess their performance, we calculated the optimum loading of these OSDAs in the 18 competing zeolites along with the target zeolite STF and prepared the aluminosilicate models. We then calculated their *E*_syn_’s for the 18 competing zeolites and STF to evaluate the stability of each zeolite-OSDA combination.

None of the OSDAs tested resulted in STF being the most stable phase according to *E*_syn_’s. Instead, AEI and CHA consistently appeared as the most stable phases for the majority of OSDAs (AEI for 19 OSDAs and CHA for 38 OSDAs). The remaining cases included 17 OSDAs favoring BEC and 4 favoring *BEA as their most stable phase. This can be explained by the fact that zeolites capable of accommodating more OSDAs per T atom tend to incorporate more aluminum content. In a previous study, we demonstrated that zeolites with higher aluminum content exhibit more favorable synthesis energies, leading to increased stability.^49^ The loading for CHA is usually 3 OSDA per unit cell (36 T atoms), which corresponds to a Si/Al ratio of 11. Similarly, in AEI, the loading is usually 4 OSDAs per unit cell (48 T atoms) which again corresponds to a Si/Al = 11. Whereas, for STF, the optimum loading of OSDAs is usually 4 molecules per unit cell (64 T atoms) leading to a Si/Al ratio of 15. This explains why AEI and CHA, which can accommodate more aluminum, showed lower *E*_syn_’s and greater stability compared to STF. This suggests that for aluminosilicate phases van der Waals stabilization energies are in general less dominant than OSDA packing and Al incorporation.

Nevertheless, we identified 8 OSDAs (5 from “actives” and 3 from “inactives”) for which *E*_syn_’s for STF belong to the top 3 most stable zeolites among the competitors. Although STF is not the most stable phase for these OSDAs, their relatively favorable *E*_syn_’s (details are given in Supporting Information, Table S14 in Section S7) suggest that they may still be viable candidates for experimental STF synthesis.

##### 4.2.1.2 OSDAs Not in Papers

To explore whether any promising candidates for STF exist within this untested space, we applied a machine learning-based QSAR (Quantitative Structure–Activity Relationship) approach to predict their potential as STF-directing agents.

Classification models were trained on a dataset of STF and non-STF OSDAs using molecular descriptors including volume, moment of inertia along the three axis vectors (Ia, Ib, Ic), and Rayleigh charge. Among these, volume and Ic emerged as the most significant predictors. The initial classification showed high sensitivity but limited specificity; however, introducing a volume filter (175–208 Å^3^) improved the accuracy of correctly identifying non-STF OSDAs, increasing specificity from around 58% to 81%. A more detailed description of the models and the descriptors is given in the Supporting Information (Section S6).

Neural network-based models were also employed using the same descriptor set but with different training strategies. One model achieved high specificity (∼99%) but low sensitivity (∼21%), effectively filtering inactive compounds while being conservative on true positives. Another model, trained with a subset of chemically similar OSDAs, improved the sensitivity–specificity balance. External validation showed this model could retain specificity while better recognizing STF-capable OSDAs. A screening of 580 untested OSDAs was performed, and candidates were selected based on two criteria: being classified as active by at least two models and having a volume within the defined range. These OSDAs are reported in Table S15 as potential STF-directing agents.

To evaluate the viability of these machine learning-predicted candidates, we computed the optimum loading of each selected OSDA in STF and its 18 competing zeolite phases, prepared the corresponding aluminosilicate models, and calculated the resulting synthesis energies. Details are included in Supporting Information (Section S7). Five OSDAs for which STF ranked among the top three most stable zeolite phases were identified.

#### 4.2.2 Search of OSDAs from OSDB for IFR

After the competing phases for IFR were selected, we evaluated potential OSDAs for IFR synthesis using a methodology applied to the entire OSDB. To identify promising candidates, we employed the shoebox algorithm,^59^ a geometry-based filtering technique that compares the molecular dimensions of candidate OSDAs to those OSDAs known to direct to IFR. Twelve OSDAs were identified to have similar shoebox dimensions with the reference sets. These OSDAs whose dimensions fit within this shoebox space were retained for further analysis.

For each OSDA, the optimum loading was calculated for IFR and its competing zeolite phases. Then, aluminosilicate models were generated and the corresponding *E*_syn_’s of each OSDA–zeolite combination were calculated. The goal was to determine whether any of these OSDAs could favor IFR as one of the most stable phases. Only one of them resulted in IFR appearing among the top three most stable frameworks, following AEI and CHA, which again exhibited more favorable *E*_syn_ values due to their ability to accommodate a higher number of OSDAs and incorporate more aluminum atoms, leading to lower (more favorable) *E*_syn_.

### 4.3 Design and Search of OSDAs for STF and IFR

For STF, novel OSDAs were designed using stochastic virtual combinatorial chemistry and de novo design. A combinatorial chemistry run was carried out based upon the reaction scheme depicted in Figure S1. The highest scoring molecule found in this run is shown in the first row of Table S4.

A second set of novel OSDAs were obtained by a run of the Synopsis de novo design algorithm. The highest scoring molecule found in this run is depicted in the second row of Table S4. The proposed reaction scheme for generating this molecule is shown in Figure S2. It may be noted that among the 1983 designed OSDAs generated during the de novo design run, several showed predicted stabilization energies that were comparable to or even more favorable than those of known OSDAs for STF. From this set, those located close to the Pareto front (∓ 0.2 kJ/mol Si) in the E–V plot (Figure 3) were selected for further *E*_syn_ calculations, following the procedure described in “Strategy-1”.

In total, 18 OSDAs that were close to the Pareto front for STF were selected for *E*_syn_ calculations, considering 19 competing zeolite phases. None of these OSDAs resulted in STF being among the top three most stable phases based on their calculated *E*_syn_’s. This suggests that, although they have favorable van der Waals interactions with STF, their packing within the aluminosilicate framework is insufficient to compete with phases like AEI and CHA under hydroxide conditions. In other words, the corresponding loading for STF leads to a Si/Al ratio larger than that for AEI and CHA, which in turn results in lower *E*_syn_’s for AEI and CHA.

For IFR, novel OSDAs were generated using the multiple stochastic combinatorial design algorithm described in the methods section using the synthesis tree depicted in Figure 4. The algorithm was run using the score function shown in Table S3 until 10000 molecules were generated, of which 3226 passed the filters and were subjected to stabilization energy calculation. The two OSDAs with the highest scores based on their stabilization energies and molecular volumes are listed in Table S5, and their suggested synthesis schemes are illustrated in Figure S3. Among the OSDAs that were subjected to stabilization energy calculations, the three highest scoring molecules were selected for *E*_syn_ calculations based on their stabilization scores and shoebox dimensions that matched those of known OSDAs used for IFR synthesis, as described in the previous section. In addition, three more OSDAs were manually designed as IFR candidates. One was based on quinuclidinium, a known structure-directing agent for AlSi-IFR, while the other two were modified versions of QG001780, one of the promising OSDAs generated by the multiple combinatorial chemistry algorithm. All six OSDA candidates for the synthesis of AlSi-IFR were evaluated using *E*_syn_ calculations against 22 competing zeolite phases. Among them, four OSDAs had IFR among their top three most stable phases, marking them as candidates for experimental testing. These OSDAs are listed in Table S17.

### 4.4 Final Choice of OSDA for STF and IFR

The final selection of OSDAs (Figures 4 and 5) was guided by both the computed synthesis energies and the practical feasibility of their preparation. For STF, among eight candidate OSDAs taken from papers (Table S14) and five hypothetical OSDA candidates (Table S15), only one OSDA was finally chosen (QHGWEBIGEQBZPY, Table S14), as its amine precursors were commercially available and it had previously been synthesized by Shvets et al.,^70^ confirming its accessibility and stability in hydroxide medium.

For IFR, from one OSDA from OSDB (Table S16) and four designed candidates (Table S17), two structurally related OSDAs derived from the same parent compound were synthesized (QG001780m and QG001780m2, Table S17). They combined low predicted *E*_syn_ values with straightforward and inexpensive synthesis routes, offering the best compromise between computational favorability and experimental feasibility.

These selected OSDAs (Table 2) were synthesized and subsequently employed in hydrothermal experiments to attempt the crystallization of Al-STF and Al-IFR. These synthesis attempts are discussed in Section 4.5.

**Table 2:**
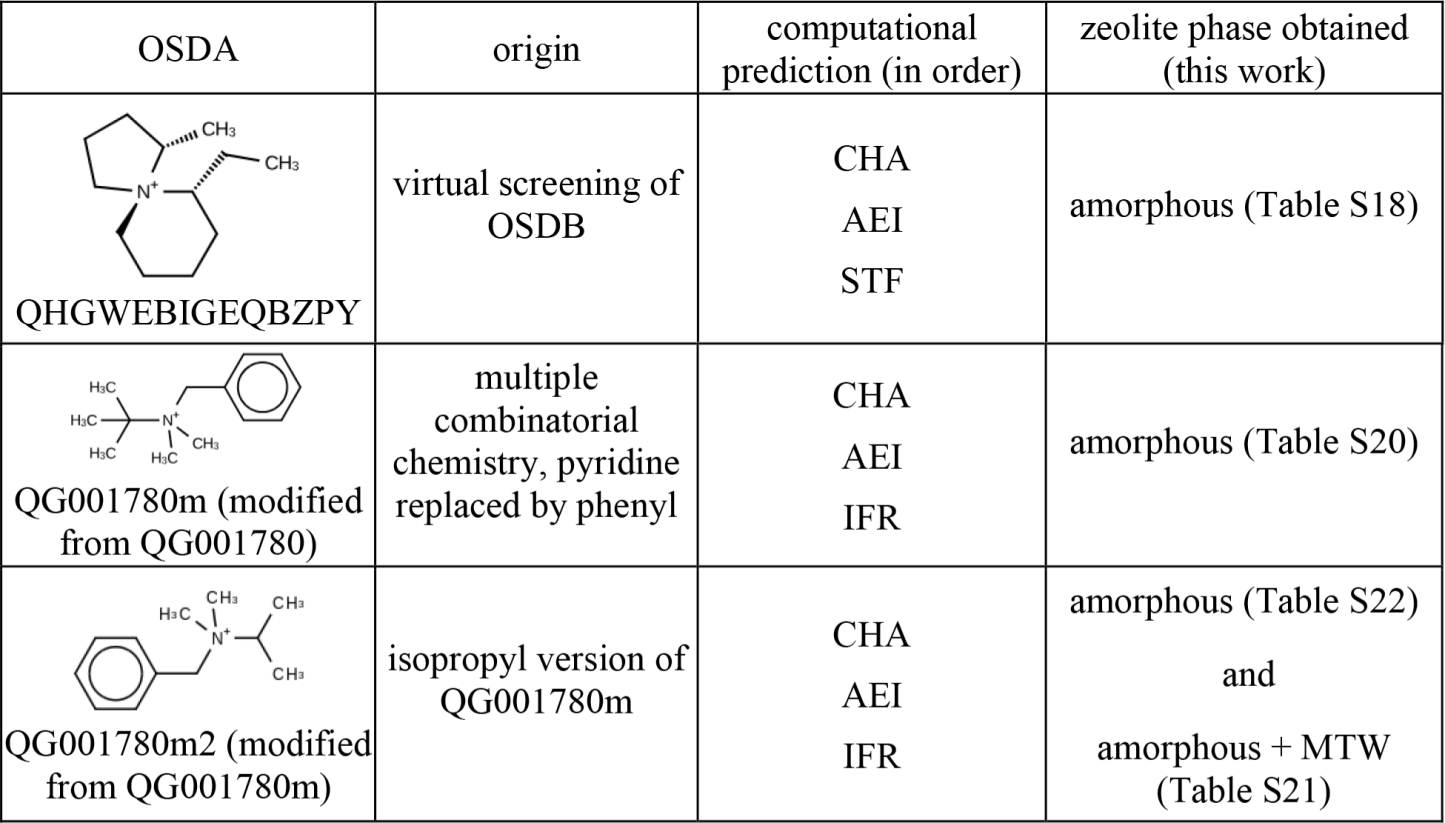
OSDAs Synthesized and Results of Aluminosilicate Zeolite Synthesis, from Gels with Composition SiO_2_: *x* Al_2_O_3_: 0.5 OSDA­(OH): 10 H_2_O, with 0 < *x* < 0.05

### 4.5 Discussion

We have analyzed more than 1000 known OSDAs from OSDB and over 7000 designed OSDAs using computational strategies, to evaluate their potential to direct the synthesis of AlSi-STF and AlSi-IFR zeolites through a combination of stabilization and *E*_syn_ calculations. To systematically identify promising OSDAs for STF and IFR zeolites, we applied two complementary workflows based on the availability of prior synthesis data for each target. For STF, which has been synthesized with a relatively large number of OSDAs as a pure silica composition, we followed Strategy 1. We employed the Pareto front that appears in the plot of stabilization energy versus molecular volume (Figure 3). OSDAs that appeared close to this front were considered good candidates and were evaluated further using *E*_syn_ calculations across STF and 18 competing zeolite phases. We then selected those for which STF ranked among the top three most stable phases, resulting in 8 promising OSDAs from the literature. In parallel, we screened the unreported or “not in papers” subset from OSDB using machine learning models trained to distinguish between STF-directing and nondirecting OSDAs. This helped us identify a smaller set of candidates, which we also subjected to *E*_syn_ calculations. From this group, 5 OSDAs were found to favor STF in the top three and were added to the final selection. These results are summarized in Section S7 of the Supporting Information, with Table S14 listing the OSDAs from papers and Table S15 those predicted through the machine learning approach. From the final pool of candidates, we assessed the practicality of synthesizing each OSDA and selected the one that was most feasible to prepare using available methods and reagents. This OSDA (QHGWEBIGEQBZPY) was synthesized and tested in a hydrothermal synthesis experiment targeting AlSi-STF. The structure of the selected OSDA and the corresponding experimental results are presented in Table 2.

For IFR, due to the limited number of known OSDAs, we applied Strategy 2, which uses the shoebox algorithm based on the geometric dimensions of known IFR-directing OSDAs. The entire OSDB database was screened using this approach to identify molecules with similar size and shape characteristics. The selected candidates were then evaluated through *E*_syn_ calculations across IFR and its competing phases. From this, one OSDA from OSDB was found to favor IFR as one of the top three most stable phases. In addition to the database screening, we also examined a set of generated OSDAs, created using stochastic virtual combinatorial chemistry and de novo design methods. These molecules were first passed through the same shoebox filtering step and then evaluated using *E*_syn_ calculations. This process led to the identification of four additional candidates that showed IFR among the most stable outcomes. These IFR candidates are presented in Section S7 of the Supporting Information, with Table S16 listing the OSDA from OSDB and Table S17 showing those obtained through molecular design strategies. From these candidates, we evaluated the feasibility of synthesizing each OSDA and selected two OSDAs, QG001780m and QG001780m2, which were prepared and both used in hydrothermal synthesis experiments targeting AlSi-IFR. These OSDAs are structural derivatives of the same parent compound, QG001780. The selected OSDAs and the outcomes of the synthesis are presented in Table 2.

Despite the careful selection of candidate OSDAs based on computational predictions, the experimental outcomes did not align with expectations. Hydrothermal synthesis attempts were carried out using QHGWEBIGEQBZPY for STF and both QG001780m and QG001780m2 for IFR, employing aluminosilicate gels in hydroxide media and in the absence of inorganic cations. The synthesis using QHGWEBIGEQBZPY resulted in the formation of an amorphous phase.

For IFR, the use of QG001780m also led to an amorphous product, whereas QG001780m2 yielded a mixture of amorphous material and MTW, which is far from being a top-rank prediction (ranked 13th), rather than the targeted aluminosilicate IFR zeolite. More details are given in Section S9.

The results of this work highlight the current limitations of computational approaches in predicting zeolite synthesis outcomes. Although methods such as the calculation of synthesis energy (*E*_syn_) provide valuable insights into the thermodynamic relation between an OSDA and a framework, they cannot fully capture the complex chemistry of crystallization in hydroxide media. Factors such as the stability of the OSDA under hydrothermal conditions and the kinetics of nucleation are not yet represented in these models.

A key lesson from this study is that not all zeolite topologies can be synthesized as aluminosilicates in hydroxide media, even when computational screening suggests a favorable host–guest interaction. The main driving force of the zeolite synthesis in aluminosilicate composition is not the short-range zeolite-OSDA interaction, but rather the ability of the zeolite micropore to pack a large charge density and promote frameworks with higher aluminum content (lower Si/Al ratio), which leads to an increased thermodynamic stability of the system. Frameworks that are stable in fluoride or pure-silica systems may remain inaccessible in hydroxide environments due to differences in charge compensation and solvation.

Moreover, our experimental observations from ^13^C and ^1^H NMR spectra of OSDAs confirm that all three OSDAs used in the zeolite synthesis were degraded or structurally modified during synthesis, which prevented the intended structure-directing effect. This helps explain why the computational predictions did not lead to successful crystallization of the target structures (aluminosilicate STF and IFR) and highlights the need for more comprehensive modeling approaches that also take into account OSDA stability and the specific chemistry of the synthesis media.

## 5 Conclusions

Our calculations suggested that it is intrinsically difficult to obtain STF and IFR as aluminosilicates in hydroxide media, with competing phases having lower predicted values of *E*_syn_. While the synthesis of pure silica zeolites is considerably driven by the strength of the zeolite-OSDA van der Waals interactions, in the case of aluminosilicate composition, the larger is the number of OSDA cations (per Si+Al atom) than can be “packed” in the micropores, the smaller becomes the resulting Si/Al ratio. Along with previous publications in our group it was found that the lower predicted values of *E*_syn_ correlate with decreasing values of Si/Al ratio and hence a larger Al content contributes to the overall stability of the system.

The difficulty in obtaining aluminosilicate STF and IFR was observed in the calculations of all ca. 600 OSDAs reported in the literature according to OSDB, along with an additional 580 unpublished (designed) OSDAs included in the OSDB, and also when designing candidate OSDAs based on quaternary ammonium structures using a software tool introduced in this work.

Nevertheless, three OSDAs were selected for which STF or IFR appeared among the top three most stable phases, and these were synthesized and tested in zeolite synthesis experiments. However, the experimental results showed no formation of either aluminosilicate STF or IFR under hydroxide conditions. While the failure to obtain STF is in line with the lack of reported successful syntheses in hydroxide media, the result for IFR suggests that successful crystallization may require very specific conditions, as it has only been synthesized in the presence of benzylquinuclidinium cations.^71^ The successful synthesis of AlSi-IFR in the presence of fluoride^72^ can be attributed to the stabilizing effect of zeolite-fluoride and fluoride-OSDA interactions in combination with the *bea* cavities ([4^3^5^2^6]) in IFR. If AlSi-STF and AlSi-IFR are to be useful in an industrial setting, their synthesis in hydroxide media is much more convenient, due to the well- known environmental problems of fluoride media, and hence the predictions in fluoride media have not been the subject of this study.

Our calculations did not model the presence of defects that may change the order of zeolite stability, but a recent computational study^32^ indicates that charge compensation of a positively charged OSDA by Al is energetically more favorable than the presence of defects. Hence, when there is sufficient Al in the synthesis gel, the number of defects in aluminosilicate zeolites is expected to be small, even in hydroxide media.

For the OSDAs we designed and synthesized, CHA and AEI were the frameworks with lower *E*_syn_. However, our experiments did not result in AEI or CHA being synthesized (neither STF nor IFR), and hence in this sense our predictions from calculations must be considered a failure.

Predicting zeolite synthesis outcomes remains an exceptionally difficult task because of the many thermodynamic, kinetic, and chemical factors involved in crystallization. This work represents a step forward by applying the *E*_syn_ descriptor to predict and experimentally test challenging synthesis targets whose preparation as aluminosilicates in hydroxide media is known to be difficult. In our previous studies, we were able to explain the phase selectivity of aluminosilicate zeolites using the *E*_syn_ descriptor. In this work, we extended the same approach to two challenging aluminosilicate systems, Al-STF and Al-IFR, which remain difficult to crystallize under hydroxide conditions. The results provide insight into why these frameworks are so hard to obtain and suggest ways to design more effective synthesis strategies in the future.

In future studies, we aim to make phase prediction more reliable by including solvent effects, especially how water and counter-cations influence framework stability and the interaction between OSDAs and the zeolite structure. We also plan to examine OSDA stability under hydrothermal conditions, as degradation or structural changes during synthesis can prevent them from guiding the formation of the desired framework. Although developing a fully comprehensive predictive model remains a long-term goal, the present results already show the importance of linking computational insight with experimental feedback to better understand and guide zeolite crystallization.

## Supplementary Material



## Notes

Synthesis energies of aluminosilicate zeolites; zeodock algorithm to calculate zeolite-OSDA van der Waals interaction; generation of quaternary ammonium OSDAs; force field simulation details; search for OSDAs for STF from “OSDAs in paper” in OSDB; machine learning AI-driven QSAR strategy; candidate OSDAs for STF and IFR according to synthesis energies; preparation of organic structure-directing agents (OSDAs); and synthesis of zeolites (PDF)

## Abbreviations

1B23DMI: 1-benzyl-2,3-dimethylimidazolium
1B3MI: 1-benzyl-3-methylimidazolium
1D: one-dimensional
AI: artificial intelligence
AlSi: aluminosilicate
ANN: artificial neural network
*E*_syn_: synthesis energy
LDA: linear discriminant analysis
LEM: lattice energy minimization
MC: Monte Carlo
ML: machine learning
OSDA: organic structure-directing agent
OSDB: Organic Structure DataBase
QSAR: Quantitative Structure–Activity Relationship
T atom: tetrahedral atom
