# Supplementary material for: Lessons from Failed Attempts of Computationally Guided Synthesis of Aluminosilicate STF and IFR Zeolites in Hydroxide Media
Source: Chem Mater. 2025 Dec 9;37(24):9689–702. doi: 10.1021/acs.chemmater.5c01751 (PMC12747119; doi:10.1021/acs.chemmater.5c01751)
Supplement: Supplementary file 1 [file cm5c01751_si_001.pdf]

Supporting Information

Lessons from failed attempts of computationally guided synthesis of aluminosilicate STF and IFR zeolites in hydroxide media

Omer F. Altundal[1], Maria Galvez-Llompart[2], Angel Cantin[1], José Valero[1], Susana Valencia[1], Fernando Rey[1], Kingsley Christian Kemp[3], Suk Bong Hong[3], Frits Daeyaert[4]*, German Sastre[1]*

[1] Instituto de Tecnología Química UPV-CSIC, Universidad Politécnica de Valencia, 46022, Valencia, Spain

[2] Molecular Topology and Drug Design Unit. Faculty of Pharmacy and Food Sciences, Department of Preventive Medicine and Public Health, Food Sciences, Toxicology and Forensic Medicine, University of Valencia, 46100, Valencia, Spain

[3] Center for Ordered Nanoporous Materials Synthesis, Division of Environmental Science and Engineering, POSTECH, Pohang 37673, South Korea

[4] SynopsisDeNovoDesign, 2340, Beerse, Belgium

Corresponding Author

* Email: gsastre@itq.upv.es, Phone: +34963879445; frits@synopsisdenovodesign.be

Contents

S1. Synthesis energies of Aluminosilicate Zeolites

S2. zeodock algorithm to calculate zeolite-OSDA van der Waals interaction

S3. Generation of quaternary ammonium OSDAs

S4. Force Field Simulation Details

S5. Search for OSDAs for STF from 'OSDAs in paper' in OSDB

S6. Machine Learning AI-Driven QSAR Strategy

S7. Candidate OSDAs for STF and IFR according to synthesis energies

S8. Preparation of organic structure-directing agents (OSDAs)

S9. Synthesis of zeolites

Section S1. Synthesis Energies of Aluminosilicate Zeolites

Table S1 presents data obtained from our previous publication[1] which contain synthesis energies for various aluminosilicate zeolites calculated with the same OSDAs but different Si/Al ratios. The data clearly show that zeolites with lower Si/Al ratios consistently exhibit more negative (more favorable) synthesis energies. This trend reflects the thermodynamic preference for incorporating Al into the framework compared to Si, since the substitution of Si^{4+} by Al^{3+} reduces the overall energy of the structure. Additionally, phases with lower Si/Al tend to accommodate more OSDAs per unit cell, which increases the packing efficiency and further stabilizes the framework.

Table S1. Synthesis energies of aluminosilicate zeolites.[1] Green and Red highlighted indicate that the computational predictions do and do not (respectively) correspond to the experimental results. SDA1-SDA7 as indicated in ref. 1.

OSDA	Exp. Zeo	Zeo	Total Energy Si/Al (eV/TO_2)	E(syn) Si/Al (eV/TO_2)	Si/Al
SDA1	CHA	BEA	-40.183	-0.535	15
		CHA	-40.109	-0.742	11
		DDR	-40.368	-0.327	29
		DOH	-40.395	-0.301	33
		MTW	-40.363	-0.354	27
		SGT	-40.228	-0.580	15
SDA2	AEI	AEI	-39.999	-0.683	11
		SFF	-40.135	-0.526	15
SDA3	AEI, CHA	AEI	-40.182	-0.795	11
		CHA	-40.188	-0.801	11
		MTW	-40.399	-0.382	27
SDA4	RTH	ITW	-40.053	-1.022	11
		RTH	-39.727	-1.426	7
SDA5	RTH	RTH	-39.647	-1.426	7
		STW	-39.775	-1.101	9
SDA6	FER	FER	-39.869	-1.261	8
		ITW	-40.073	-0.994	11
		TON	-40.274	-0.490	23
SDA7		EUO	-40.262	-0.718	13
		ITH	-40.285	-0.741	13
		MRE	-40.411	-0.473	23

Section S2. zeodock algorithm to calculate zeolite-OSDA van der Waals interaction

The zeodock algorithm has been developed to efficiently and reproducibly estimate the stabilization energy of an OSDA into an all-silica zeolite framework. The algorithm is composed of four steps: random placement of single OSDA copies into the zeolite unit cell, selection of tuples of OSDA copies that do not overlap with one another, rigid optimization of the VDW energies of the selected tuples in rotation-translation space, and frozen pose molecular mechanics energy minimization of the OSDA-zeolite complexes.

Before starting the docking algorithm, the 3D structure of the OSDA has to be generated. This can be a single conformation of the OSDA, or a set of low energy conformers. For OSDAs with rotatable bonds, a genetic algorithm to generate all locally minimized low energy conformers in torsion space within an energy gap of 1 kcal/mol from the lowest energy conformation is used. A description of this algorithm can be found in ref 2. In addition, for molecules with unsaturated rings, a ring flipping algorithm is used to generate multiple ring conformations as input for the conformational search in torsion space.

The four steps of the zeodock algorithm are discussed below.

To fit single copies of the OSDA into a zeolite framework, a rectangular grid comprising the unit cell is defined, with a grid spacing of 0.1 Å. Grid points within a threshold distance of 3.3 Å of an atomic position in the unit cell are removed. In a loop, a large number, typically 1E6, of OSDA copies in a random orientation are positioned at randomly picked remaining grid points. For flexible OSDAs, a conformation from the previously determined set of low energy conformations is selected at random. For each pose, the sum of the overlaps between van der Waals radii of the OSDA and zeolite atoms is calculated. The overlap between individual atom pairs is calculated as the difference between the sum of the van der Waals radii of the atoms, scaled by a factor of 0.9, and the interatomic distance. When this difference is negative it is clipped to zero. During the loop, the N poses, typically 1000, with the lowest overlap are retained. If N poses with zero overlap are obtained before the 1E6 trials, the loop exits. Poses with an overlap larger than a threshold, by default 1. Å, see below, are rejected. When no poses are retained after applying this threshold, the zeodock algorithm decides that the OSDA does not fit into the framework.

In the second step, to dock multiple copies of an OSDA into a zeolite framework, combinations of single copies obtained in the previous step are chosen such that no overlap between the copies occurs. The overlap between the copies is calculated in the same way as the OSDA-zeolite overlap. The number of copies has to be specified before running this step in the algorithm. For more than two copies and large numbers of single-copy poses, the number of combinations becomes impossible to screen exhaustively and a genetic algorithm (GA) is applied to find non-overlapping combinations of OSDA copies. The chromosomes of the GA are combinations of indices of the single copies obtained in the first step of the algorithm, and mutation, crossover and selection operators are applied to evolve a population of initial random combinations. Chromosomes encoding identical OSDA poses are not allowed to enter the population. The fitness of a chromosome is the overlap between the OSDA copies defined by the indices plus the sum of the individual OSDA-zeolite overlaps that have been calculated in

the first step of the algorithm. The population size is typically 250. The algorithm is halted when all chromosomes in the population have zero overlap, or when a maximum number of function evaluations, typically 1E6, have been performed. The final population of the GA defines a set of poses defined by tuples of OSDA copies with OSDA-zeolite overlap less than the maximum overlap allowed in the first step of the zeodock algorithm, and a minimal OSDA-OSDA overlap. Poses with a total overlap larger than a threshold, typically 5 Å, see below, are discarded. When no poses are retained, the zeodock algorithm decides that the given number of OSDA copies does not fit into the framework.

The third step in the docking algorithm is a local optimization in translation-rotation space of the OSDA-zeolite poses obtained in the previous step. The energy minimized is the sum of the OSDA- zeolite and OSDA-OSDA van der Waals interaction energies in the zeolite framework unit cell, with periodic boundary conditions applied. The orientation of the rigid OSDA copies is defined by their Euler angles, the energy is minimized using a BFGS minimizer until the rms gradient is below a threshold. A threshold energy is set to select the optimized poses that enter the next step. The value of this threshold energy depends on the zeolite framework, see below. When no poses with an energy below the threshold value are obtained, the zeodock algorithm decides that the given number of OSDA copies does not fit into the framework.

The final step of the docking algorithm is a molecular mechanics optimization of the OSDA-zeolite poses obtained in the previous step. The stabilization energy of the OSDA in the framework is determined using the frozen-pose method as described in ref 3. The N lowest energy complexes, with N typically 25, obtained in the previous step are energy minimized with periodic boundary conditions and with the volume of the unit cell held constant. The force field used is the Dreiding force field.[4] Stabilization energies are obtained as the differences between the energies of the minimized complexes and the energies of the zeolite and OSDA structures extracted from the minimized complexes.[3] The energy differences are divided by the number of Si atoms in the zeolite framework to obtain the stabilization energy in units of kJ/(mol Si). The complex with the lowest stabilization energy is output as the result of the zeodock algorithm.

The algorithm depends on a number of meta parameters listed in Table S2. Default values of most parameters were obtained by benchmarking on known OSDA-zeolite pairs, seeking a compromise between reproducibility of the end result and computational efficiency. The maximum number of OSDA copies that fit into a given target framework has to be determined by trial and error. The overlap thresholds used in steps one and two may be tightened or relaxed based upon information from experimental data on known OSDA-zeolite pairs. The threshold energy in step three depends on the target framework and is determined from the energies obtained from known OSDAs. Application of this threshold is important, as the input of highly strained OSDA-zeolite complexes into the next step may produce highly distorted zeolite structures with a misleadingly low predicted stabilization energy.

Table S2. Meta parameters of the zeodock algorithm.

step	Parameter	Default Value
1	Grid spacing	0.1 Å
	grid threshold distance	3.3 Å
	trial poses	1E6
	scale factor for VDW radii	0.9
	overlap threshold	1. Å
	poses retained	1000
2	OSDA copies	framework dependent
	GA population size	250
	GA function evaluations	1E6
	overlap threshold	5 Å
3	threshold energy	framework dependent
4	complexes minimized	25

Section S3. Generation of quaternary ammonium OSDAs: stochastic virtual combinatorial chemistry, de novo design, stochastic multiple combinatorial chemistry

Stochastic virtual combinatorial chemistry was performed using the Reopt program.[5] The reaction scheme used to generate quaternary ammonium OSDAs is shown in Figure S1. The scoring function used to evaluate the virtual reaction products is summarized in Table S3. As reagents, 2215 secondary amines and 781 acid chlorides were compiled from ChemSpace[6] to participate in the first reaction step towards a tertiary amine. For the final reaction step, 106 halogens were compiled to generate a quaternary ammonium by reacting with the tertiary amine formed in the second reaction step. The chemical search space therefore consisted of ~1.8×10^8 compounds. The algorithm was run until a total number of 10000 OSDAs were evaluated, 846 of which passed the molecular filters in Table S3 and were subjected to the calculation of the stabilization energy in STF.

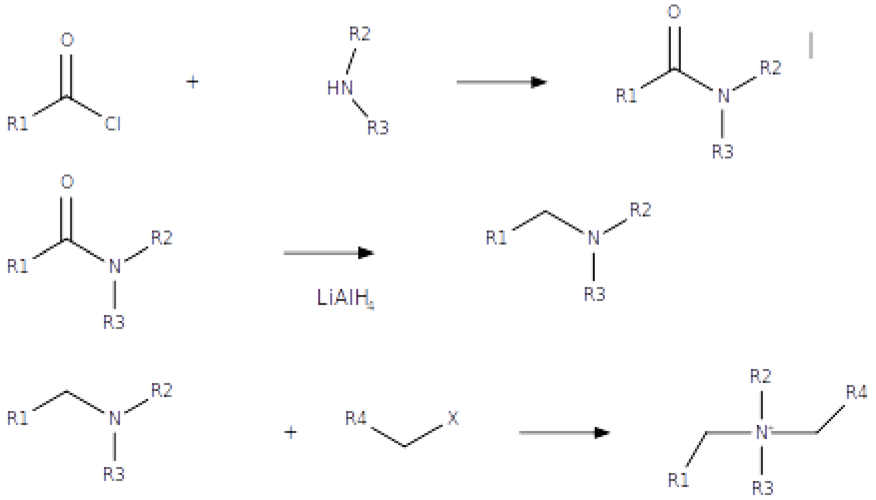

Figure S1. Reaction scheme used in stochastic virtual combinatorial chemistry to generate quaternary ammonium SDAs.

Table S3. Scoring function used to design OSDS directed towards STF and IFR.

Score	Score Type	Thresholds
Molecular Mechanics Compatibility	Binary	
SP3 Rotatable Bonds	Smaller than threshold	3
Total Rotatable Bonds	Smaller than threshold	6
Volume	Bracketed	0-400Å
C, N or H only	Smaller than threshold	0
Unwanted Functional Groups	Smaller than threshold	0
C to N+ ratio	Smaller than threshold	18
Stabilization Energy	Minimize	

De novo design was performed using the Synopsis program described in ref 7. The same multi objective scoring function was used as with the de novo design (Table S3). The presence of a quaternary ammonium functionality is enforced by defining the C to N+ ratio as one of the scores. The chemical search space was defined by a set of 101 reactions and a database of 7635 commercially available reagents with a listed purchase price of < 100 USD per gram. With the maximum number of synthesis steps set to 5, the estimated size of the chemical search space is 3.5×10^{20} molecules. The algorithm was run until a total number of 10000 OSDAs were evaluated, 1137 of which passed the molecular filters and were subjected to the calculation of the stabilization energy in STF.

Table S4. Highest scoring putative OSDAs targeting STF found by stochastic combinatorial chemistry and de novo design. These OSDAs were subjected to synthesis energy calculations along with other candidate OSDAs as explained in Strategy 1 (Figure 4).

Method	molecule	Volume (Å^3)	C to N+ ratio	stab. energy (kJ/mol Si)
stochastic combinatorial chemistry	Syn007758 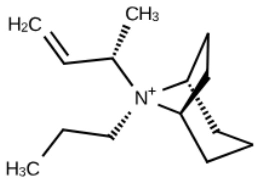	220	14.0	-14.2
de novo design	Syn002675 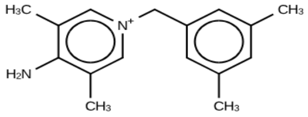	238	16.0	-16.0

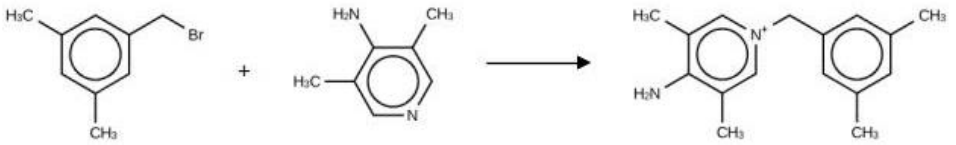

Figure S2. Proposed synthesis route of the highest scoring de novo designed STF OSDA.

Table S5. Highest scoring putative OSDAs targeting IFR found by multiple combinatorial chemistry. These OSDAs were subjected to synthesis energy calculations along with other candidate OSDAs as explained in Strategy 2 (Figure 5).

molecule	Volume (Å^3)	C to N+ ratio	stab. energy (kJ/mol Si)
QG001777 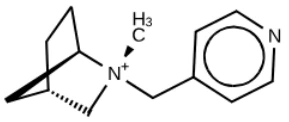	198	13.0	-15.1
QG001780 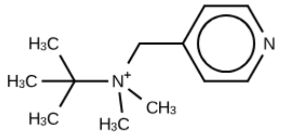	200	12.0	-15.0

Figure S3. Proposed synthesis routes for the highest scoring putative OSDAs targeting IFR found by multiple combinatorial chemistry.

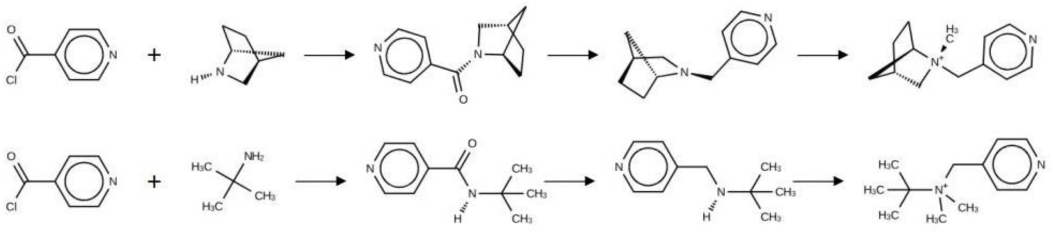

Section S4. Force field simulation details

To determine the optimal loading and conformation of OSDAs within zeolite pores, we employed the zeoTsda software.[8] In order to do that, zeoTsda software utilizes the General Utility Lattice Program (GULP)[9] to perform a series of Monte Carla simulations and lattice energy minimizations. After identifying the optimal loading for all zeo-OSDA systems, we utilized zeoTAl software,[10] to generate various Al distributions, following Loewenstein's rule, for each zeo-OSDA pair. The aim was to ensure that the charge from the incorporated Al species balanced the charges of the OSDAs in each system. The generated Al distributions were then geometry-optimized, and the one with the lowest total energy was selected as the optimal distribution for each zeo-OSDA system.

In the simulations, Lennard-Jones potential was employed to model the non-bonded two-body interactions between all atoms in the system with a cut-off radius of 12 Å. Potential parameters of the OSDA atoms was generated using the method of Oie et al.[11] whereas the potential parameters of framework atoms were taken from the force field of Bushuev and Sastre (BS).[12] In the simulations, electrostatic interactions were defined by Coulombic potential.[13] Ewald summation was used to incorporate long-range electrostatic interactions.[14] The SDA molecules were assigned a cationic charge, usually +1, to compensate for the anionic charges from Al-(O_4)-$(SiO_{3/2})_4$ units. A charge equilibration method was employed to determine the charge distribution of SDAs, along with $Si(OH)_4$, $Al(OH)_3$, and H_2O molecules.[15] BS force field was used to designate the charges of atoms in the zeolite framework, where the charges for Si and O atoms in the central SiO_4 tetrahedra of Si-(O_4)-$(SiO_{3/2})_4$ units were 2.1 and −1.05, respectively, ensuring a total charge of zero. For the central AlO_4 tetrahedra in Al-(O_4)-$(SiO_{3/2})_4$ units, the charges of Al and O were 1.575 and −1.16875, respectively, resulting in an overall charge of −1 $(1.575 - 1.16875\times4 + 2.1\times4 - 1.05\times6)$.

Section S5. Search for OSDAs for STF from 'OSDAs in paper' in OSDB

To search for candidate OSDAs for STF from the OSDB, we identified the OSDAs that are used to synthesize pure silica STF from OSDB and called these OSDAs 'active'. We then found that all active OSDAs were closely grouped along a straight-line trend in the stabilization energy versus volume graph (Figure 2). This line shows the Pareto front, representing the optimal balance between stabilization energy and molecular volume for synthesizing STF. When we examined the data of all OSDAs in the E-V plot, we identified 42 OSDAs in the database that were classified as inactive, although they fell within ±0.2 kJ/mol Si of this linear trend. We labeled these OSDAs as 'inactive-close' OSDAs since they do not synthesize STF but have similar properties than that of the active OSDAs (36 OSDAs). Since, these OSDAs are close to the Pareto front, we classified these OSDAs as potential candidate OSDAs, along with active OSDAs, for synthesizing aluminosilicate STF and performed synthesis energy simulations for them. The list is given in Table S6.

Table S6. Inchikey and SMILES representations of active and inactive-close OSDAs for STF taken from Figure 2, that includes data from 'OSDAs in papers' subset of OSDB database.

InChIKey	SMILES
AAVHECBNSNZYLZ-NXEZZACHNA-N	CC[N@+]1(C)CCCC[C@H]1C
AAVHECBNSNZYLZ-VHSXEESVNA-N	CC[N@+]1(C)CCCC[C@@H]1C
AKLZASLFLGDNMF-GFCCVEGCNA-N	CC[C@@H]1CCCC[N+]12CCCCC2
AMTHKVRAVJJVJT-CMPLNLGQNA-N	CC1(C)[C@H]2CC[C@]1(C)C[N+](C)(C)C2
CAMAYIGCVWBIQO-SNVBAGLBNA-N	CC(C)[C@H]1CCCC[N+]1(C)C
CBQITPWSTPVHCM-JQWIXIFHNA-N	CC1(C)C[C@H]2C[C@@](C)(C1)C[N+]2(C)C
CBQITPWSTPVHCM-ZYHUDNBSNA-N	CC1(C)C[C@@H]2C[C@](C)(C1)C[N+]2(C)C
CGQMGLUHXVEXSG-RNJOBUHINA-N	C[N+]1(C)C[C@@H]2CCC[C@H]1[C@@H]1CC[C@H]2C1
CGQMGLUHXVEXSG-UMSGYPCINA-N	C[N+]1(C)C[C@H]2CCC[C@@H]1[C@@H]1CC[C@H]2C1
CGQMGLUHXVEXSG-YVECIDJPNA-N	C[N+]1(C)C[C@H]2CCC[C@@H]1[C@H]1CC[C@@H]2C1
CWEGCQIIDCZZED-UHFFFAOYNA-N	C1CCC(CN2CCCC2)CC1
CYWPTQCQTBNBAP-UHFFFAOYNA-N	CC1N(C)CC[N+]1CCC[N+]1CCN(C)C1C
DMBILWVUAAPKHW-FOPUMGAHNA-N	C[N+](C)(C)[C@H]1[C@@H]2C[C@H]3C[C@@H](C2)C[C@@H]1C3
DXVZBAYWVZNZGE-UWVGGRQHNA-N	C[N+]1(C)CCC[C@@H]2CCC[C@@H]21
DXVZBAYWVZNZGE-VHSXEESVNA-N	C[N+]1(C)CCC[C@@H]2CCC[C@H]21
DXVZBAYWVZNZGE-ZJUUUORDNA-N	C[N+]1(C)CCC[C@H]2CCC[C@@H]21
FMYWBUTUSOGTAE-UWVGGRQHNA-N	C[N+]1(C)CCC[C@H]2CC[C@H]1C2
FMYWBUTUSOGTAE-VHSXEESVNA-N	C[N+]1(C)CCC[C@H]2CC[C@@H]1C2
FMYWBUTUSOGTAE-ZJUUUORDNA-N	C[N+]1(C)CCC[C@@H]2CC[C@H]1C2
FXJTWOUGULWIMG-DCAQKATONA-N	C[C@H]1CC[N+](C)(C)[C@H]2C[C@@H]1C2(C)C
FXJTWOUGULWIMG-GMTAPVOTNA-N	C[C@@H]1CC[N+](C)(C)[C@@H]2C[C@H]1C2(C)C
FXJTWOUGULWIMG-VWYCJHECNA-N	C[C@@H]1CC[N+](C)(C)[C@H]2C[C@@H]1C2(C)C
GORHIJLTGHKCCV-VHSXEESVNA-N	C[N+]1(C)C[C@@H]2C[C@H](C1)[N+](C)(C)C2
HGACHMQVWWZPCX-DTORHVGONA-N	C[C@@H]1C[C@H](C)C[N+](C)(C)C1
HGACHMQVWWZPCX-RKDXNWHRNA-N	C[C@@H]1C[C@@H](C)C[N+](C)(C)C1
JQQUXIDNUKATMZ-ATZCPNFKNA-N	C[C@H]1C[N@@+]2(C)CCN1C[C@@H]2C
JQQUXIDNUKATMZ-FXPVBKGRNA-N	C[C@@H]1C[N@@+]2(C)CCN1C[C@H]2C
JQQUXIDNUKATMZ-NGZCFLSTNA-N	C[C@@H]1CN2CC[N@@+]1(C)C[C@@H]2C
JQQUXIDNUKATMZ-WCABBAIRNA-N	C[C@@H]1C[N@@+]2(C)CCN1C[C@@H]2C
JQQUXIDNUKATMZ-YWVKMMECNA-N	C[C@@H]1C[N@+]2(C)CCN1C[C@@H]2C
KWGRBVOPPLSCSI-WPRPVWTQNA-N	CN[C@@H](C)[C@H](O)C1CCCCC1
LVTXTVWKBOOKOQ-GHMZBOCLNA-N	C[N+]1(C)CCC[C@H]2CCCC[C@H]21

LVTXTVWKBOOKOQ-QWRGUYRKNA-N	C[N+]1(C)CCC[C@@H]2CCCC[C@@H]21
MDWZIHPBIGDFDH-UHFFFAOYNA-N	C[N+]12CCCC3CCCC(C31)CCC2
MNHFOWPOCZTFTR-AOOOYVTPNA-N	CN1C[C@H]2C[C@@H](C1)C[N+](C)(C)C2
MPAWGVZMQBYYPT-IJLUTSLNNA-N	C[C@@H]1C[C@H]2CC[C@]1(C)CC[N+]2(C)C
MPAWGVZMQBYYPT-QJPTWQEYNA-N	C[C@H]1C[C@H]2CC[C@]1(C)CC[N+]2(C)C
NMKGZHREQMSRQP-CHWFTXMANA-N	C[N+]1(C)C[C@H]2CC[C@@H]1[C@H]1CC[C@@H]2O1
NMKGZHREQMSRQP-DBIOUOCHNA-N	C[N+]1(C)C[C@H]2CC[C@@H]1[C@@H]1CC[C@H]2O1
NOROEQADYUDNFL-UHFFFAOYNA-N	CC(C)[C]1N(C)C=CN1C
OTCBTFQRGQSVNM-ZYANWLCNNA-N	C[N+]1(C)CC2C[C@H]3CC1C[C@@H](C2)C3
OXFHNKYFIVWAAT-UHFFFAOYNA-N	CC[C]1N(C)C=CN1C
PGJCYFGAKJUZEN-USZNOCQGNA-N	C[N+]1(C)C[C@@H]2CC[C@H]1[C@@H]1CC[C@H]2C1
PLABWEYBNUBTTM-NXEZZACHNA-N	CC[N+]1(C)C[C@H](C)C[C@@H](C)C1
PLABWEYBNUBTTM-UWVGGRQHNA-N	CC[N+]1(C)C[C@@H](C)C[C@H](C)C1
PLIVCIVEQZKZJI-LLVKDONJNA-N	C[C@@H]1CCC[N+]12CCCCCC2
PUTPMTKFVUXVQX-GHMZBOCLNA-N	C[C@@H]1CCC[C@@H](C)[N+]12CCCC2
PUTPMTKFVUXVQX-QWRGUYRKNA-N	C[C@H]1CCC[C@H](C)[N+]12CCCC2
QAFMBNSVYCPGTF-SECBINFHNA-N	CC[C@@H]1CCCC[N+]1(C)C
QHGWEBIGEQBZPY-RWMBFGLXNA-N	CC[C@H]1CCCC[N@+]12CCC[C@@H]2C
QHGWEBIGEQBZPY-YNEHKIRRNA-N	CC[C@@H]1CCCC[N@+]12CCC[C@@H]2C
QJKOQOZWQZPIKD-NXEZZACHNA-N	CC[N+]1(CC)C[C@@H](C)O[C@H](C)C1
QOURMEHOWGLDIZ-JGPRNRPPNA-N	CC[N@+]1(C)[C@H](C)CCC[C@@H]1C
QOURMEHOWGLDIZ-URLYPYJENA-N	CC[N@@+]1(C)[C@H](C)CCC[C@@H]1C
QUPNWQBGKWGRPI-GHMZBOCLNA-N	CC[N+]1(CC)CCC[C@@H](C)[C@H]1C
QUPNWQBGKWGRPI-MNOVXSKENA-N	CC[N+]1(CC)CCC[C@@H](C)[C@@H]1C
SDXMPRCSJWHJRH-BKUVIOGVNA-N	C[N+]1(C)C[C@@H]2[C@H](C1)[C@@H]1C=C[C@H]2CC1
SDXMPRCSJWHJRH-IWDIQUIJNA-N	C[N+]1(C)C[C@@H]2[C@H](C1)[C@H]1C=C[C@@H]2CC1
SZWHXXNVLACKBV-UHFFFAOYNA-N	CC[P](CC)(CC)CC
UIQHUHPIRRYLDE-GHMZBOCLNA-N	CC[C@@H]1CCCC[N@@+]1(C)CC
UIRYAWVSBUZMBF-LBPRGKRZNA-N	CC(C)[N@@+]1(C)CCCC(C)(C)C1
VLRHDZUUSAKSTQ-VHSXEESVNA-N	C[N+]1(C)CC[C@@H]2CCC[C@@H]2C1
VLRHDZUUSAKSTQ-ZJUUUORDNA-N	C[N+]1(C)CC[C@H]2CCC[C@H]2C1
WCWCWFXJYPMQGR-RYUDHWBXNA-N	C[C@H]1CCC[C@H](C)[N+]12CCCCC2
WTKZWCLKCKVWNQ-ZJUUUORDNA-N	CCN1C[C@H]2C[C@@H](C1)[N+](C)(C)C2
XEYHFNNNWRPOHF-UHFFFAOYNA-N	C1CC[N+]2(CC1)CCCC2
XPLXACTVVUDCNQ-UHFFFAOYNA-N	CC[N+](CC)(CC)C(C)C
XTMMQPIKFURNRR-NEPJUHHUNA-N	C[N+]1(C)CCC[C@H]2CCCC[C@H]2C1
XULIXFLCVXWHRF-UHFFFAOYNA-N	CN1C(C)(C)CCCC1(C)C
XYEVWVBQYOGNMF-UHFFFAOYNA-N	CC(C)[P+](C)(C(C)C)C(C)C
YRGRAYWIIXQMRX-AOOOYVTPNA-N	C[N+]1(C)C[C@H]2CC[C@H](CC2)C1
YVGRXHOOIZGRMJ-UHFFFAOYNA-N	CC[C]1N(CC)C=C(C)N1CC
YWZGBJRUJMCHLS-GHMZBOCLNA-N	CC[N+]1(CC)[C@H](C)CCC[C@H]1C
ZRKPEQRQHQPWIE-UHFFFAOYNA-N	C1CC[N+]23CCCCC2(C1)CCCC3
ZRYCQACEYYTRHK-CYDGBPFRNA-N	C[C@H]1[C@H](C)[C@@]2(C)CC[C@@H]1[N+](C)(C)CC2
ZRYCQACEYYTRHK-FDYHWXHSNA-N	C[C@@H]1[C@@H](C)[C@]2(C)CC[C@H]1[N+](C)(C)CC2
ZYOMXQGIWAKIBK-DTORHVGONA-N	C[C@@H]1CCC[C@H](C)[N+]1(C)C
ZYOMXQGIWAKIBK-IUCAKERBNA-N	C[C@H]1CCC[C@H](C)[N+]1(C)C

Section S6. Machine learning AI-driven QSAR strategy

A machine learning AI-driven QSAR strategy has been employed to infer the potential activity of organic compounds in directing the synthesis towards STF zeolite. For this purpose, three models have been trained using linear discriminant analysis (Model #1) and artificial neural networks (Models #2 and #3).

The first model, based on a discriminant function (DF), was developed using Linear Discriminant Analysis (LDA) to classify OSDAs according to their capacity to direct STF zeolite synthesis. This model was trained on a dataset of 453 OSDAs, comprising 24 STF-directing OSDAs and 429 non-STF OSDAs (Table S7). The analysis produced a Wilks' lambda of 0.972, with an associated F-statistic of 6.526 and a p value of 0.0016, indicating that the discriminant functions were statistically significant.

$DF\_STF=(V\times0.017)-(Ic\times0.001)-1.984$ Model #1

$N= 453;\quad \lambda=0.972;\quad F=6.526;\quad p=0.0016$

Table S7. Classification matrix for Model #1.

	Classification accuracy (%)	Classified as STF OSDA	Classified as Non-STF OSDA
**Train set**			
STF OSDA	92	22	2
Non-STF OSDA	58	179	250
**Test set**			
STF OSDA	88	7	1
Non-STF OSDA	55	74	89

The classification accuracy from the LDA model (Table S7) reveals that Model #1 is significantly more sensitive than specific, a pattern consistent across both the training and test set of compounds.

Of the eight descriptors used to characterize the OSDAs, the forward stepwise procedure based on p-values identified *Volume* and *Ic* as the most significant variables for distinguishing between OSDAs capable of directing STF zeolite synthesis and those that are not. The volume of an organic molecule is a critical factor in its capacity to function as an OSDA, as it influences the molecule's ability to occupy the cavities and channels of the developing zeolite framework. An appropriate volume ensures that the OSDA can shape and stabilize the intended structure, A suitable volume ensures that the OSDA can properly fit within these cavities, thereby guiding the growth and stabilization of the target structure. This, in turn, facilitates the effective synthesis of the desired zeolite.

Upon analysis of the data, a distinct pattern in the Volume descriptor emerges for STF OSDAs, with volume values ranging from 175 to 208. By incorporating both the DF_{STF} criterion and

the Volume descriptor, the model's predictive accuracy is markedly enhanced in identifying OSDAs that do not promote STF synthesis. As presented in Table S8, the classification accuracy improves from 58% to 81% in the training set and from 55% to 79% in the test set when this additional criterion is applied. Furthermore, the classification performance becomes more balanced with respect to both sensitivity and specificity.

Table S8. Classification matrix considering both criteria: DF and volume value.

	**Classification accuracy (%)**	**Classified as STF OSDA**	**Classified as Non-STF OSDA**
**Train set**			
**STF OSDA**	**92**	**22**	**2**
**Non-STF OSDA**	**81**	**82**	**347**
**Test set**			
**STF OSDA**	**88**	**7**	**1**
**Non-STF OSDA**	**79**	**34**	**129**

The second type of models used in this computational study are AI (artificial intelligence) models employing neural networks. The ANN models (Models #2 and #3) were trained to identify OSDAs with and without the capacity to direct the formation of STF zeolite. In these models, we will adopt a slightly different strategy by training the networks using all available descriptors: heavies, V, Ia, Ib, Ic, Ray, eps and kappa.

Model #2 consists of a multilayer perceptron (MLP) artificial neural network, which includes one input layer, one hidden layer, and one output layer. The input layer comprises eight neurons, corresponding to the eight descriptors used to characterize the OSDAs. The hidden layer consists of three neurons, while the output layer contains two neurons, representing the two groups into which the network classifies the OSDAs: those that direct STF synthesis and those that do not.

Model #2 achieved a high average accuracy rate of 95% in classifying molecules in both the training (N=368) and internal validation sets (N=85) (see Table S10). The algorithm used to train the network is BFGS (Broyden-Fletcher-Goldfarb-Shanno) method, which in this case required 19 iterations to converge and reach a solution for the classification problem. To calculate the error associated with the classifications made by the neural network, the entropy function is employed. The hyperbolic tangent function served as the activation function connecting the input layer neurons to the hidden layer neurons, while the softmax function was used to connect the hidden layer neurons to the output layer, assigning probabilities to the two categories in our model. Both functions are non-linear activation functions.

Table S9. Architecture of ANN Model #2: Training and Validation Performance, Training Algorithm, and Activation Functions Used in Both Hidden and Output Layers.

ANN Model #2	Training perf. (%)	Validation perf. (%)	Training algorithm	Error function	Hidden activation	Output activation
MLP 8-3-2	95	95	BFGS 19	Entropy	Tanh	Softmax

When evaluating the model's classification performance, it is important to highlight its high specificity, achieving 99% accuracy for Non-STF OSDAs in the training data (N=349 OSDAs). However, as shown in Table S10, while the model effectively identifies OSDAs that do not direct STF formation, it recognizes only a small percentage of those that successfully lead to STF synthesis, achieving only 21% accuracy for STF OSDAs. Consequently, when applied this AI-model to a small dataset of organic compounds, the model may be overly strict. Conversely, it could excel at screening millions of compounds due to its high rate of detecting false active compounds.

Table S10. Classification matrix for Model #2.

	Classification accuracy (%)	Classified as STF OSDA	Classified as Non-STF OSDA
**Train set**			
STF OSDA	21	4	15
Non-STF OSDA	99	3	346
**Validation set**			
STF OSDA	20	1	4
Non-STF OSDA	100	0	80

In Table S10, the classification matrix generated by the internal validation set (N=85) for Model #2 is also presented. As expected, it reflects the same pattern exhibited by the training set, achieving 100% accuracy in the category of non-STF directing OSDAs, while only achieving 20% accuracy in the category of OSDAs that direct STF zeolite formation.

Finally, we conducted an external validation of the network (N=171). The external set exhibits the same pattern as the training and validation sets, demonstrating high specificity with 98% accuracy classification for non-STF OSDAs. However, as expected, the model was unable to recognize any STF OSDAs (N=8).

The third model is an artificial neural network (ANN), which differs from Model #2 in its selection of OSDAs used for training. In this case, a limited dataset comprising 63 OSDAs was selected: all available OSDAs known to direct STF synthesis were assigned to the active

category, whereas the inactive category included OSDAs that do not promote STF zeolite formation but share chemical similarity with the active OSDAs.

Model #3 achieved a relatively low average accuracy rate of 60% on the training set (N=63), while exhibiting a considerably higher accuracy rate on the internal validation sets (N=14) (Table S11). The network was trained using the BFGS algorithm, which required 20 iterations to converge and reach a solution for the classification problem. To estimate the error associated with the neural network's classifications, the entropy function was employed. The mathematical function connecting the input layer neurons to the hidden layer neurons was the exponential function, whereas the softmax function connected the hidden layer neurons to the output layer. Both activation functions are nonlinear in nature.

Table S11. Architecture of ANN Model #3: Training and Validation Performance, Training Algorithm, and Activation Functions Used in both Hidden and Output Layers.

ANN Model #3	Training perf. (%)	Validation perf. (%)	Training algorithm	Error function	Hidden activation	Output activation
**MLP 8-5-2**	**60**	**79**	**BFGS 20**	**Entropy**	**Exponential**	**Softmax**

In Table S12, it could be seen Model #3's classification accuracy. This model shows a greater specificity (74% of inactive OSDA correctly classified – N=35) than sensitivity (42% of STF OSDA correctly classified-N=28). However, this pattern is not shared by the internal validation set of compounds achieving 75% correct classification for active OSDA (N=4) and 80% accuracy for Non-STF OSDA (N=10).

Table S12. Classification matrix from Model #3.

	Classification accuracy (%)	Classified as STF OSDA	Classified as Non-STF OSDA
**Train set**			
**STF OSDA**	**42**	**12**	**16**
**Non-STF OSDA**	**74**	**9**	**26**
**Validation set**			
**STF OSDA**	**75**	**3**	**1**
**Non-STF OSDA**	**80**	**2**	**8**

Given that Model #3 was trained on a dataset with lower chemical diversity, conducting an external validation test was essential. The external test set comprised exclusively OSDAs that do not direct STF synthesis, as all STF-active OSDAs had been included in the ANN training phase. Model #3 successfully classified 80% of the external test set (436 out of 547). This

modeling strategy demonstrates that ANN Model #3 was able to overcome the lack of sensitivity observed in ANN Model #2 without compromising specificity.

Computational virtual screening strategy

A computational virtual screening strategy based on the three trained and validated models was conducted to identify potential STF OSDAs. The criteria for labeling compounds as potential STF OSDAs were: (a) classification as active by at least two of the three models, and (b) a volume ranging from 175 to 208 Å³. A set of 580 OSDAs with unknown activity regarding their ability to direct STF formation was screened. Detailed information on the screening classification is omitted here for brevity but can be obtained from the authors upon request. Table S13 provides the list of OSDAs that meet the established criteria for potential STF activity.

Table S13. List of potential OSDA (**InChIKey** format) leading to STF zeolite, including descriptors values, DF_{STF} values, classification labels from Models #1, #2 and #3, and classification probabilities.

**Compound**	**Model #1 LDA**					**Model #2 ANN**		**Model #3 ANN**	
	V	Ic	DF_{STF}	Cl.	P.A.	Cl.	C.L.	Cl.	C.L.
AFJDLJMCNOGZFT-UHFFFAOYNA-N	177.902	735.533	0.229	STF	0.561	No STF	0.948	STF	0.502
AFJDLJMCNOGZFT-ZSBIGDGJNA-N	177.884	735.765	0.228	STF	0.561	No STF	0.948	STF	0.502
HEJVKSJBFVLVBD-MDZLAQPJNA-N	208.259	947.587	0.503	STF	0.624	No STF	0.948	STF	0.509
HMUPHBSEYMDRSS-AAEUAGOBNA-N	193.79	933.821	0.267	STF	0.570	No STF	0.948	STF	0.604
HMUPHBSEYMDRSS-DGCLKSJQNA-N	193.796	933.888	0.267	STF	0.570	No STF	0.948	STF	0.603
HMUPHBSEYMDRSS-WCQYABFANA-N	193.749	863.563	0.351	STF	0.590	No STF	0.948	STF	0.692
HMUPHBSEYMDRSS-YPMHNXCENA-N	193.761	863.723	0.351	STF	0.590	No STF	0.948	STF	0.693
HMUPHBSEYMDRSS-YUZLPWPTNA-N	193.798	933.95	0.267	STF	0.570	No STF	0.948	STF	0.604
IQJLOSSCAGCHAM-IJLUTSLNNA-N	194.26	902.021	0.314	STF	0.581	No STF	0.948	STF	0.738
JFLLRCKFJNJNTA-FOSCPWQONA-N	186.788	868.265	0.224	STF	0.560	No STF	0.948	STF	0.523
JFLLRCKFJNJNTA-GHMZBOCLNA-N	186.633	784.81	0.322	STF	0.583	No STF	0.948	STF	0.526
JFLLRCKFJNJNTA-QWRGUYRKNA-N	186.625	784.204	0.322	STF	0.583	No STF	0.948	STF	0.525
KKSYTWWCLLRDPE-PHIMTYICNA-N	177.973	661.346	0.319	STF	0.582	No STF	0.866	STF	0.504
MPAWGVZMQBYYPT-SRVKXCTJNA-N	192.285	783.604	0.421	STF	0.606	No STF	0.948	STF	0.526
MPAWGVZMQBYYPT-WOPDTQHZNA-N	192.294	817.184	0.381	STF	0.597	No STF	0.948	STF	0.532
MWIQRNSGQAOEMQ-RNJOBUHINA-N	193.024	784.132	0.434	STF	0.608	No STF	0.948	STF	0.517
MWIQRNSGQAOEMQ-SDDRHHMPNA-N	193.248	768.664	0.456	STF	0.613	No STF	0.948	STF	0.546

MWIQRNSGQAOEMQ-UTUOFQBUNA-N	193.268	768.419	0.457	STF	0.614	No STF	0.948	STF	0.547
OWUFMQFWIHWPKF-PHIMTYICNA-N	179.142	917.689	0.032	STF	0.514	No STF	0.948	STF	0.517
OWUFMQFWIHWPKF-QWRGUYRKNA-N	179.002	832.807	0.131	STF	0.538	No STF	0.948	STF	0.534
PLIVCIVEQZKZJI-NSHDSACANA-N	178.774	810.284	0.154	STF	0.543	No STF	0.948	STF	0.543
QHGWEBIGEQBZPY-FRRDWIJNNA-N	193.968	889.899	0.323	STF	0.583	No STF	0.948	STF	0.618
QHGWEBIGEQBZPY-XQQFMLRXNA-N	193.953	889.613	0.323	STF	0.583	No STF	0.948	STF	0.617
QJKOQOZWQZPIKD-UWVGGRQHNA-N	180.282	870.759	0.108	STF	0.533	No STF	0.948	STF	0.599
QUPNWQBGKWGRPI-QWRGUYRKNA-N	186.369	818.958	0.276	STF	0.572	No STF	0.948	STF	0.547
QUPNWQBGKWGRPI-WDEREUQCNA-N	186.532	773.696	0.333	STF	0.585	No STF	0.948	STF	0.529
RPNTWZAQIBHWGT-UHFFFAOYNA-N	192.942	793.19	0.421	STF	0.606	No STF	0.948	STF	0.590
RPNTWZAQIBHWGT-ZSBIGDGJNA-N	192.965	793.264	0.422	STF	0.606	No STF	0.948	STF	0.591
ULBFMCWPNOTKNP-LLVKDONJNA-N	178.772	846.026	0.111	STF	0.533	No STF	0.948	STF	0.509
VVUVYZZGKWEDLX-GHMZBOCLNA-N	186.777	761.575	0.352	STF	0.590	No STF	0.948	STF	0.545
VVUVYZZGKWEDLX-QWRGUYRKNA-N	186.887	819.012	0.285	STF	0.574	No STF	0.947	STF	0.516
WCWCWFXJYPMQGR-VXGBXAGGNA-N	192.881	838.128	0.366	STF	0.593	No STF	0.948	STF	0.569
ZCBWZIDLZUDXMT-XBFCOCLRNA-N	207.879	939.093	0.506	STF	0.625	No STF	0.948	STF	0.540
ZPADAZTVSGCTNF-UHFFFAOYNA-N	186.972	983.957	0.089	STF	0.528	No STF	0.948	STF	0.592

Section S7. Candidate OSDAs for STF and IFR according to synthesis energies

This section lists OSDAs for which STF or IFR appears among the top three most stable zeolite phases in hydroxide media, based on synthesis energy calculations. The candidates originate from two sources: (i) existing OSDAs from the OSDB database, and (ii) designed OSDAs generated via de novo or virtual combinatorial design approaches. These OSDAs represent the most promising computational predictions for directing the synthesis of STF or IFR under aluminosilicate conditions.

Candidate OSDAs for STF

The Active OSDAs refer to OSDAs that have been experimentally reported to direct the synthesis of STF in pure silica form. On the other hand, Inactive OSDAs have not been associated with formation of STF but were selected for analysis because they lie close to the Pareto front in the stabilization energy vs. OSDA volume plot. This proximity suggests they exhibit favorable van der Waals interactions and molecular volumes similar to known STF-directing agents, making them strong candidates under aluminosilicate conditions.

Table S14. Candidate OSDAs that contain STF in top three zeolite phases according to their synthesis energies selected from the subset 'OSDAs in paper' in OSDB. Column "SDA name" indicates the 8 final OSDAs that appear in the left-column selection process of Figure 4.

SDA name	STF in rank	(In)Active	Competing Phase 1	Competing Phase 2	ChemDraw
cgqmgluhxvexsg-rnjobuhina-n	3	Active	AEI	CHA	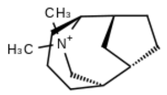
fxjtwougulwimg-gmtapvotna-n	3	Active	AEI	CHA	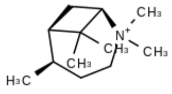
qupnwqbgkwgrpi-mnovxskena-n	3	Active	CHA	BEA	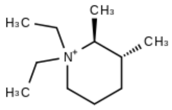
uiqhuhpirrylde-ghmzboclna-n	3	Active	CHA	IFR	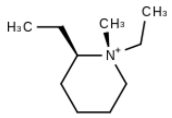
uiryawvsbuzmbf-lbprgkrzna-n	2	Active	CHA	IFR	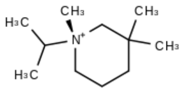
dmbilwvuaapkhw-fopumgahna-n	2	Inactive	AEI	RTH	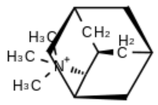
hgachmqvwwzpcx-dtorhvgona-n	3	Inactive	CHA	AEI	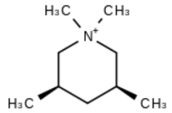
qhgwebigeqbzpy-rwmbfglxna-n	3	Inactive	CHA	AEI	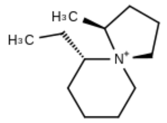

Table S15. Candidate OSDAs that contain STF in top three zeolite phases according to their synthesis energies selected from the subset 'OSDAs not in paper' in OSDB. Column 'SDA name' indicates the 5 final OSDAs that appear in the right-column selection process of Figure 4.

SDA name	STF in rank	Competing Phase 1	Competing Phase 2	ChemDraw
hejvksjbfvlvbd-mdzlaqpjna-n	3	CHA	AEI	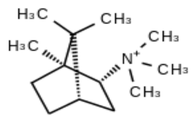
iqjlosscagcham-ijlutslnna-n	3	AEI	CHA	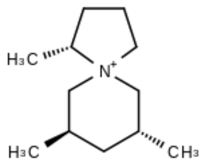
jfllrckfjnjnta-foscpwqona-n	3	AEI	CHA	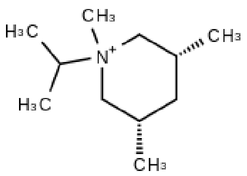
owufmqfwihwpkf-qwrguyrkna-n	3	CHA	LTA	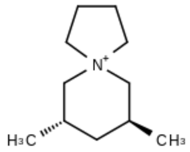
rpntwzaqibhwgt-uhfffaoyna-n	3	CHA	AEI	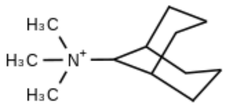

Candidate OSDAs for IFR

Table S16. Candidate OSDA that contain IFR in top three zeolite phases according to its synthesis energy selected from OSDB. Column "SDA name" indicates the final OSDA that appear in the left-column selection process of Figure 5.

SDA name	IFR in rank	Competing Phase 1	Competing Phase 2	ChemDraw
vtksrdvidrqpcp-wopdtqhzna-n	3	AEI	CHA	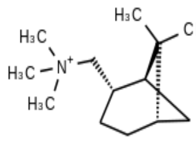

Table S17. Candidate OSDA that contain IFR in top three zeolite phases according to its synthesis energy selected from designed OSDAs. Column "SDA name" indicates the 4 final OSDAs that appear in the right-column selection process of Figure 5.

SDA name	IFR in rank	Competing Phase 1	Competing Phase 2	ChemDraw
syn000079	3	CHA	AEI	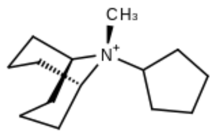
qg001780	3	CHA	AEI	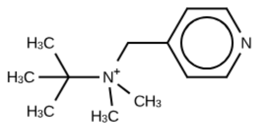
qg001780m	3	CHA	AEI	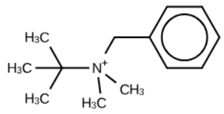
qg001780m2	3	CHA	AEI	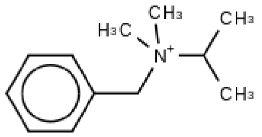

Section S8. Preparation of organic structure-directing agents (OSDAs)

Synthesis of OSDA for STF

The OSDA selected as candidate for the synthesis of STF (see Table 1) was prepared according to the general method to synthesize spirocyclic ammonium salts.[16-18]

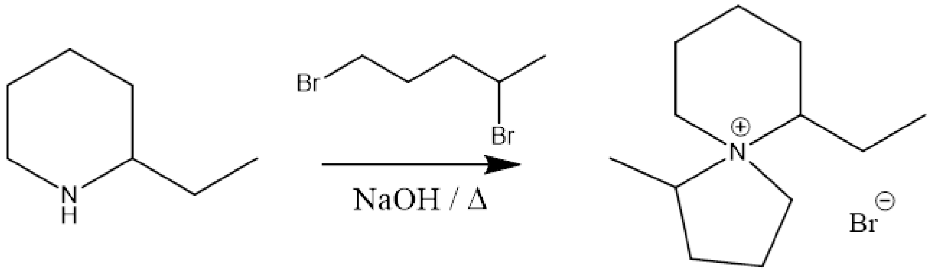

Figure S4. Synthesis of 6-ethyl-1-methyl-5-azaspiro[4.5]decan-5-ium bromide (qhgwebigeqbzpy in Tables 1 and S14).

Synthesis of the spirocyclic ammonium salt. A mixture of 43.20 g (187.72 mmol) of 1,4-dibromopentane and 250.0 mL of 1.5 M NaOH solution was heated under reflux. Then 25.0 mL (187.72 mmol) of 2-ethylpiperidine were dropped into it for a period of 2 hours. Finally the mixture was heated under reflux overnight. After cooling with an ice bath 300.0 mL of an ice-cold solution of NaOH (40%) were added. The crude was extracted three times with $CHCl_3$. All the organic extracts were dried over $MgSO_4$, filtered and partially evaporated under vacuum. Addition of diethyl ether provided a huge white precipitate which was filtered under vacuum and washed with diethyl ether obtaining 39.13 g (79%) of the different stereoisomers of the desired spirocyclic ammonium salt.

^{1}H NMR (D_2O, 400.1 MHz) d: 3.83 and 3.63 (m+m, 2 H,), 3.43 (m, 1H,), 3.01 (m, 2 H), 2.82 (m, 2 H), 2.17-1.42 (m, 5 H), 1.43-1.23 (m, 2 H), 1.22-1.01 (m, 5 H) and 0.71 (m, 6 H). ^{13}C NMR (D_2O, 10.6 MHz) d: 72.06, 71.05, 68.23, 66.48, 62.74, 57.69, 52.43, 51.23, 31.64, 27.59, 26.71, 23.01, 21.69, 20.92, 19.73, 18.69, 15.61, 11.56 and 10.57. Anal. Calcd. for $C_{13}H_{22}IN$: C, 54.96; H, 9.23; N, 5.34. Found: C, 49.24; H, 9.50; N, 5.03. See Figure S9.

Synthesis of OSDA for IFR iodide (QG001780m in Table 1)

The quaternization reaction of the proposed pyridine-derived OSDA (see Table 1) is expected to yield a dicationic species, as the pyridine ring is more prone to nucleophilic attack by the methylating agent than the amine group. To overcome this limitation, the cyclohexyl-derived organic cation was synthesized as an alternative. This modification assumes that there are no significant structural or functional differences between the proposed pyridine-OSDA and the experimentally accessible cyclohexyl-analogue, which can be obtained in high yield and is suitable for subsequent zeolite synthesis.

Synthesis of the OSDA starts with a reductive amination of benzaldehyde with *t*-butylamine using $NaBH_3CN$ as a reducing agent. Leucart's reaction over the resulting secondary amine allowed to introduce a methyl group into the N. Finally, quaternisation with CH_3I provided the ammonium salt in a iodide form.

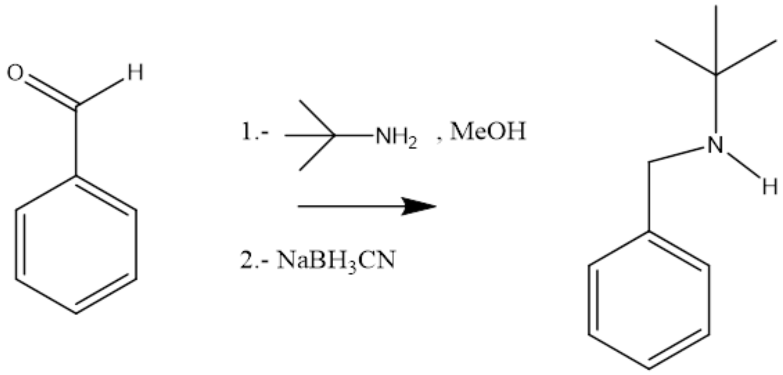

Figure S5. Synthesis of *N*-benzyl-2-methylpropan-2-amine

Reductive amination of benzaldehyde with t-butylamine. 10.02 g (94.44 mmol) of benzaldehyde and 9.9 mL (94.44 mmol) of *t*-butylamine were solved in 250.0 mL of MeOH. After 90 minutes of vigorous stirring at room temperature 7.10 g (113.33 mmol) of $NaBH_3CN$ were added. The mixture was kept under continuous stirring at room temperature overnight. At this point 235.0 mL of H_2O were added and the resulting solution was extracted three times with CH_2Cl_2. All the organic extracts were dried over $MgSO_4$, filtered and concentrated under vacuum providing 11.20 g (73%) of the desired secondary amine with satisfactory purity to use in the next step without further purification.

^1H NMR ($CDCl_3$, 400.1 MHz) d: 7.41 (m, 5H, Ar-*H*), 3.79 (s, 2H, NCH_2) and 1.26 (s, 9H, 3 CH_3). ^{13}C NMR ($CDCl_3$, 10.6 MHz) d: 141.35, 128.78, 128.56, 127.00, 50.81, 47.31 and 29.16.

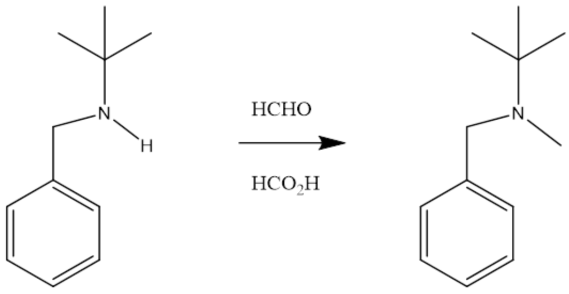

Figure S6. Synthesis of *N*-benzyl-*N*,2-dimethylpropan-2-amine

Synthesis of the tertiary amine. 11.20 g of the secondary amine were suspended in 30.0 mL of formaldehyde (37% acq. solution). 15.0 mL of formic acid were dropped into the previous suspension and the resulting mixture was heated at 100 ° C overnight. After cooling at room temperature the resulting solution was poured into 500.0 mL of a 2.0 M NaOH solution and extracted three times with $CHCl_3$. All the organic extracts were dried over $MgSO_4$, filtered and

concentrated under vacuum providing 9.78 g (80%) of the desired tertiary amine with satisfactory purity to use in the next step without further purification.

^{1}H NMR ($CDCl_3$, 400.1 MHz) d: 7.53 (m, 5H, Ar-*H*), 3.75 (s, 2H, NCH_2), 2.36 (br s, 3H, NCH_3) and 1.42 (s, 9 H, 3 CH_3). ^{13}C NMR ($CDCl_3$, 10.6 MHz) d: 141.78, 128.75, 128.30, 127.00, 55.54, 54.30, 35.00 and 26.50. See Figure S11.

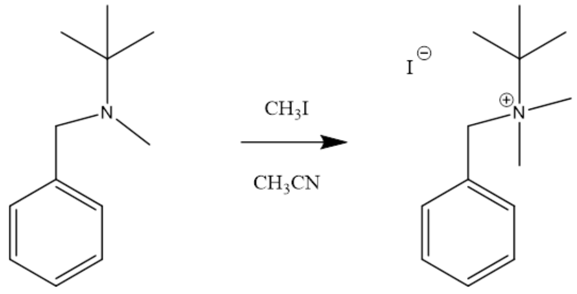

Figure S7. Synthesis ofN-benzyl-*N*,*N*,2-trimethylpropan-2-aminium iodide (qg001780m in Tables 1 and S17).

Synthesis of the ammonium iodide salt. 9.78 g (55.15 mmol) of the previously prepared amine were solved in 300.0 mL of CH_3Cl and cooled in an ice bath. Over this solution 17.1 mL (275.7 mmol) of CH_3I were dropped through an ace addition funnel. Almost immediately a white solid appeared. The mixture was kept under stirring at room temperature for 6 days. The obtained solid was filtered, washed with diethyl ether and dried under vacuum providing 13.07 g of the desired ammonium salt (46%).

^{1}H NMR (D_2O, 400.1 MHz) d: 7.48 (m, 5H, Ar-*H*), 4.39 (s, 2H, NCH_2), 2.75 (s, 6H, 2 NCH_3) and 1.53 (s, 9 H, 3 CH_3). ^{13}C NMR (D_2O, 10.6 MHz) d: 133.47, 130.45, 129.00, 127.84, 73.34, 61.77, 44.01 and 23.33. Anal. Calcd. for $C_{13}H_{22}IN$: C, 48.91; H, 6.95; N, 4.39. Found: C, 48.51; H, 7.12; N, 4.37.

Another Synthesis of OSDA for IFR

Synthesis of N-benzyl-N,N-dimethylpropan-2-aminium iodide (QG001780m2 in Table 1)

20.0 g (148 mmol) of dimethylbenzylamide (≥99 %, Aldrich) was solved into 200.0 mL of acetone and to this solution 37.7 g (0.222 mmol) of 2-iodopropane (99 %, Aldrich) was added. The resulting mixture was then heated at 80 °C for 4 days to give a white solid. The acetone was evaporated and the remaining solid washed with ether until the ether runs clear. The solid was then dried using rotary evaporation at 80 °C for 2 hours under reduced pressure. Yield: 97.2 %

^{1}H NMR (300 MHz, D_2O): δ 7.46 (m, 5H, Ar-H), 4.37 (s, 2H, CH_2), 3.65 (m, 1H, $(CH_3)_2CH$), (s, 6H, $(CH_3)_2$), 1.40 (d, 6H, $(CH_3)_2CH$) ppm. ^{13}C NMR (75 MHz, D_2O): δ 133.0, 130.6, 129.1, 127.3, 66.0, 64.8, 46.6, 16.0 ppm. see Figures S13 and S15.

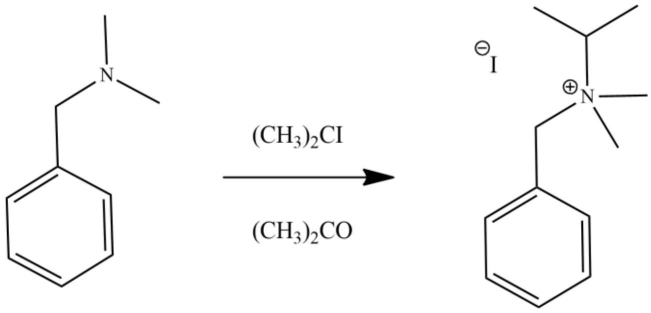

Figure S8. Synthesis of N-benzyl-N,N-dimethylpropan-2-aminium iodide.

Where necessary, the iodide form of the OSDA was converted into the hydroxide form by aqueous anion exchange using Amberlite IRN-78 anion-exchange resin (Alfa). The resulting solution was concentrated using rotary evaporation at 80 °C, and the concentration determined by titration against 0.1 M HCl with phenolphthalein as an indicator.

Section S9. Synthesis of zeolites

The materials were synthesized using the OSDAs prepared as described above, after the ionic exchange to their hydroxide forms. To do this, the halide salts were dissolved in water and submitted to ion exchange with a hydroxide resin (Amberlite IRN-78), determining the hydroxide concentration by titration with HCl (0.1 N) using phenolphthalein as an indicator.

Synthesis of materials using the OSDA selected to target STF (see Table 1)

The materials were synthesized using the OSDA from a gel of the following composition, with ‘x’ indicated in Table S18:

$$SiO_2 : x\ Al_2O_3 : 0.5\ OSDA(OH) : 10\ H_2O$$

The gels were prepared by adding tetraethylortosilicate (TEOS) (98%, Merck), as the silica source, and aluminum isopropoxide (>98%, Merck), as the aluminum source, to an aqueous solution containing 0,309 equivalents of hydroxide of OSDA per 1000 g (OSDA(OH)). The mixture was kept under stirring until the ethanol produced during hydrolysis of TEOS was evaporated and the required water content was achieved. Finally, the mixture was introduced in Teflon-lined stainless-steel autoclaves and heated at different temperatures under static conditions for several days. Then, the autoclaves were cooled down and the materials were recovered by filtration, washing with deionized water and acetone and drying at 100ºC during 5 hours. As shown in Table S19, the synthesized OSDA was obtained as a mixture of four possible stereoisomers, each with its corresponding enantiomer, resulting in eight potential configurations. This mixture fully explains the splitting observed in the NMR spectra, since the synthetic route does not selectively produce any single isomer. The OSDA (qhgwebigeqbzpy-rwmbfglxna-n) used for the computational calculations corresponds to only one of these configurations, representing approximately one-quarter of the total organic cations present. The role of the remaining isomers remains uncertain, but they are unlikely to promote crystallization exclusively toward the targeted structure. Attempts to isolate individual isomers were unsuccessful, as obtaining sufficient amounts of pure OSDA for synthesis proved unfeasible. Moreover, the ^{13}C NMR spectra of the recovered mother liquors (Figure S9) indicate that the OSDA is unstable under the applied synthesis conditions, as many of the original resonances disappear and new peaks appear at higher chemical shifts (around 160 and 127 ppm), consistent with the formation of enamine and olefinic species.

Table S18. Results of synthesis using OSDA for target STF.

Entry	Si/Al	x	Temperature (ºC)	Time (Days)	Phase
1	15	0.0333	175	15	Amorphous
				30	Amorphous
2	∞	0	175	17	Amorphous
				36	Amorphous
3	10	0.05	175	15	Amorphous
				29	Amorphous
4	10	0.05	150	15	Amorphous
				32	Amorphous
5	10[a]	0.05	150	15	Amorphous
				30	Amorphous
6	10[b]	0.05	150	15	Amorphous
				30	Amorphous
7	10	0.05	200	14	Amorphous
				28	Amorphous
8	35	0.0143	200	18	Amorphous
				50	Amorphous

a) $Al(OH)_3$ used as the aluminum source
b) $H_2O/Si = 50$

Table S19. List of different possible conformational isomers of the OSDA code qhgwebigeqbzpy-rwmbfglxna-n. For sake of clarity, only one enantiomer is show for each configuration. The third entry corresponds to the isomer used in this study for the computational calculations.

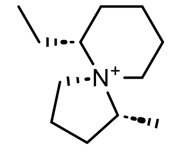	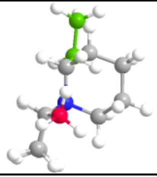
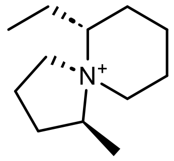	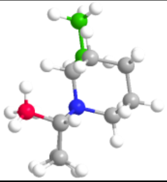
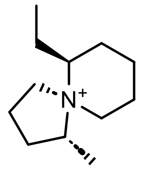	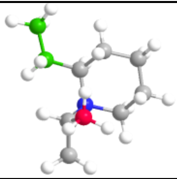
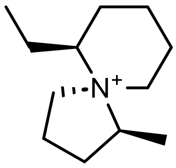	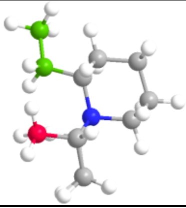

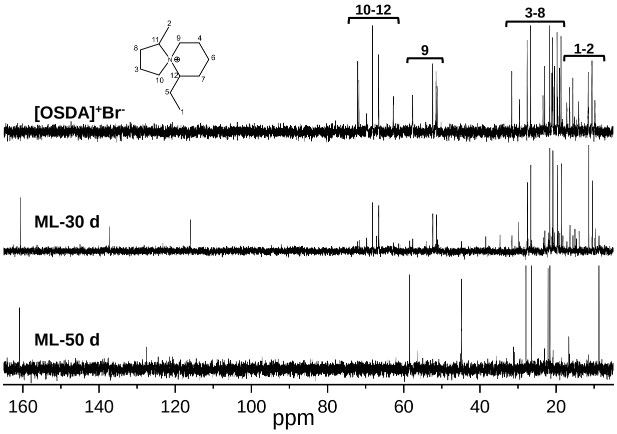

Figure S9. ^{13}C-NMR spectra of the as-made OSDA (qhgwebigeqbzpy-rwmbfglxna-n) and the corresponding mother liquors after 30 and 50 days of zeolite synthesis.

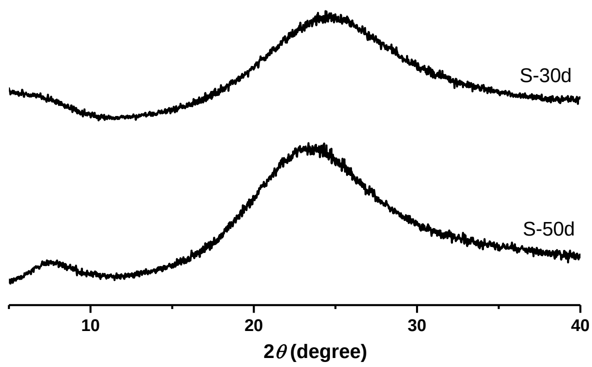

Figure S10. XRD patterns of the solid obtained in presence of OSDA (qhgwebigeqbzpy-rwmbfglxna-n) after 30 and 50 days of synthesis (Table S18, entries 6 and 8).

Synthesis of materials using the first OSDA selected to target IFR (see Table 1)

The materials were synthesized using the OSDA from a gel of the following composition, with 'x' indicated in Table S19:

$$SiO_2 : x\ Al_2O_3 : 0.5\ OSDA(OH) : 10\ H_2O$$

The gels were prepared by adding tetraethylortosilicate (TEOS) (98%, Merck), as the silica source, and aluminum isopropoxide (>98%, Merck), as the aluminum source, to an aqueous solution containing 0.265 equivalents of hydroxide of OSDA per 1000 g (OSDA(OH)). The mixture was kept under stirring until the ethanol produced during hydrolysis of TEOS was evaporated and the required water content was achieved. Finally, the mixture was introduced in Teflon-lined stainless-steel autoclaves and heated at different temperatures under static conditions for several days. Then, the autoclaves were cooled down and the materials were recovered by filtration, washing with deionized water and acetone and drying at 100 ºC during 5 hours. The OSDA, N-benzyl-N,N-trimethylpropan-2-aminium, was obtained as a pure compound, with its ^{13}C and ^{1}H NMR spectra fully consistent with the expected functional groups. However, the NMR spectra of the recovered mother liquors (Figure S11) show that the OSDA is not stable under synthesis conditions, as the characteristic resonances of the intact molecule progressively disappear with increasing crystallization time, while new peaks appear around 160 and 127 ppm, corresponding to enamine and olefinic species.

Table S20. Results of synthesis using OSDA for target IFR.

Entry	Si/Al	x	Temperature (ºC)	Time (Days)	Phase
1	15	0.0333	175	15	Amorphous
				29	Amorphous
2	15[a]	0.0333	175	15	Amorphous
				30	Amorphous
3	50	0.01	175	15	Amorphous
				30	Amorphous
4	50	0.01	200	14	Amorphous
				40	Amorphous
5	∞	0	175	28	Amorphous
				42	Amorphous
6	10[b]	0.05	175	18	Amorphous
				32	Amorphous

a) $Al(OH)_3$ used as aluminum source
b) Si/OSDA(OH) = 1

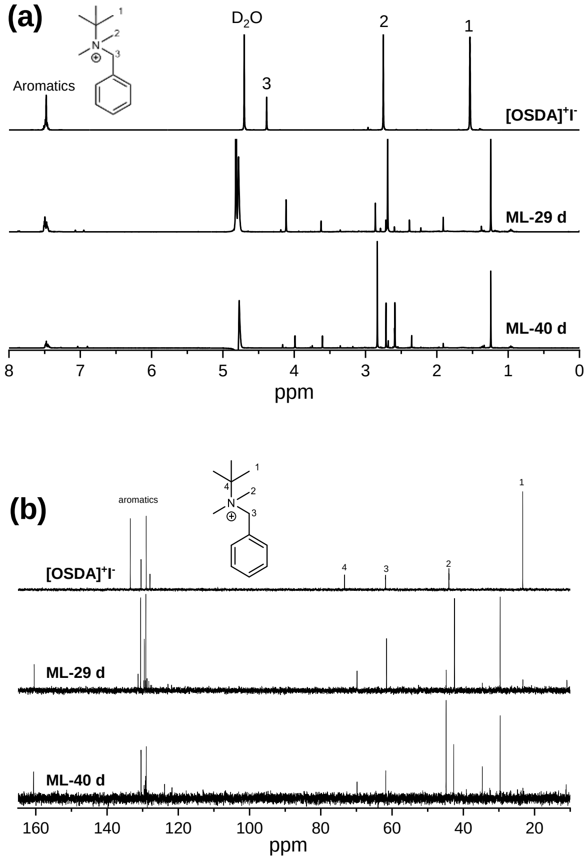

Figure S11. (a) ^{1}H NMR and (b) ^{13}C NMR and spectra of the as-made OSDA (qg001780m, N-benzyl-N,N-trimethylpropan-2-aminium) and the corresponding mother liquors after 29 and 40 days of zeolite synthesis.

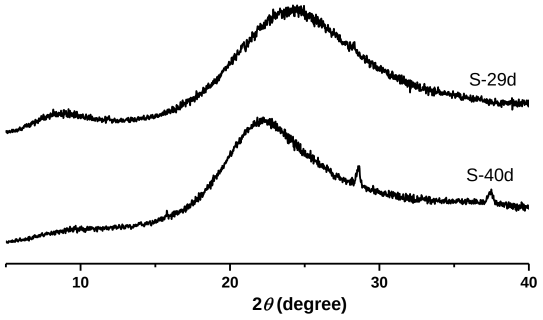

Figure S12. XRD patterns of the solid obtained in presence of OSDA (qg001780m) after 29 and 40 days of synthesis (Table S20, entries 1 and 4).

Synthesis of materials using the second OSDA selected to target IFR

The starting point for the zeolite syntheses using N-benzyl-N,N-dimethylpropan-2-aminium as an OSDA are based on those reported for SSZ-42 (IFR) and MCM-58 (IFR),[19, 20] as these methods allowed us to determine the effect of the OSDA with or without alkali metal cations during the crystallizations. The reagents employed include the N-benzyl-N,N-dimethylpropan-2-aminium OSDA prepared here (see below), fumed silica (Aerosil 200, Degussa), 30 wt % colloidal silica (Ludox AS-30, Aldrich), aluminum metal (99.0 %, Samchun), aluminum sulfate octadecahydrate (>98 %, Acros), and deionized water. To assess whether the N-benzyl-N,N-dimethylpropan-2-aminium cation was stable in the applied synthesis conditions, mother liquors were isolated and liquid ^{1}H and ^{13}C NMR spectra obtained. From the ^{1}H NMR data we were then able to determine that the amount of OSDA degradation was ca. 3 % in the presence of Na^{+} and K^{+} cations after crystallization for 4 and 1 days, respectively in Figures S13 and S15.

SSZ-42 type syntheses

The original SSZ-42 synthesis employed zeolite Y (LZY-52) as an aluminum source which limits the synthesis to those where Na is always included in the synthesis gel, furthermore this method can be considered to follow an interzeolite transformation pathway in contrast to the theoretical study. As such, we employed Al metal as a source, allowing us to study the effect of the OSDA with or without Na cations present. The materials were synthesized from gels with the following composition:

$$SiO_2 : x\ Al_2O_3 : y\ Na_2O : z\ R(OH) : 43.3\ H_2O$$

where R is the OSDA prepared, and x, y, and z are varied between 0.05 < x < 0.025, 0 < y < 0.18, and 0.30 < z < 0.66, respectively. The gels were prepared by dissolving the aluminum metal in the OSDA. To this mixture, the fumed silica, sodium hydroxide, and H_2O were added and the mixture stirred for 24 hours. Finally, the mixture was loaded into 23 mL Teflon-lined autoclaves and heated under rotation (60 rpm) at 150 or 165 °C for 14 days. The products were isolated using filtration, repeatedly washed with distilled water, and then dried at room temperature. Subsequently the Powder X-ray diffraction (PXRD) patterns were recorded on a PANalytical X'Pert diffractometer (Cu Kα radiation) with an X'Celerator detector.

Table S21. Results of synthesis using N-benzyl-N,N-dimethylpropan-2-aminium in SSZ-42 type conditions.[a]

Si/Al	Na_2O	R(OH)	Temperature (°C)	Product
10	0.18	0.3	150	ZSM-12 + A[b]
			165	MOR
20			150	MOR + A
			165	MOR + D
10	-	0.66	150	A[c]
			165	A[c]
20			150	ZSM-12 + A
			165	A[c]

[a]All syntheses were performed under rotation (60 rpm) for 14 days. [b] The product appearing first is the major phase. ‘A’ and ‘D’ indicate amorphous and dense phases, respectively. [c] Product obtained after

21 days.

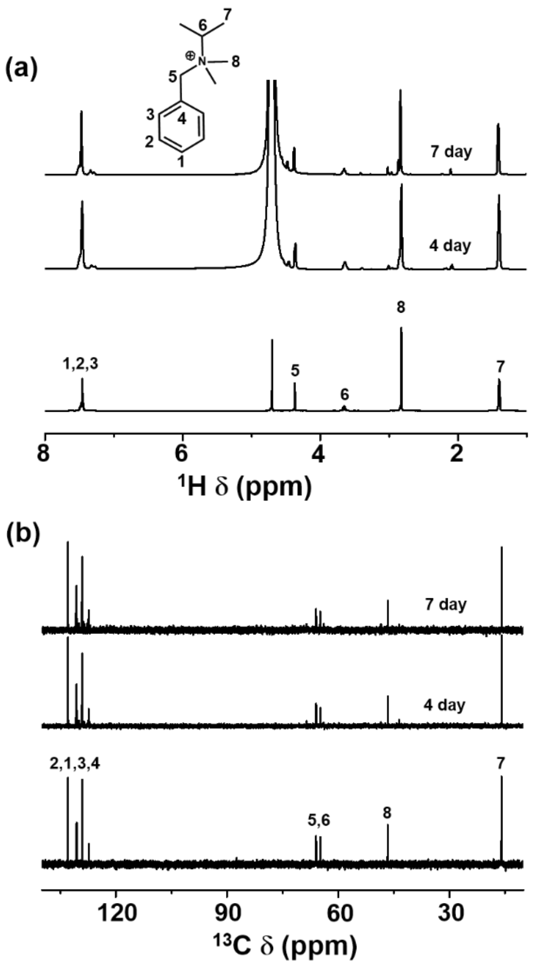

Figure S13. Liquid (a) ^{1}H NMR and (b) ^{13}C NMR of the N-benzyl-N,N-dimethylpropan-2-aminium cation obtained from mother liquors of syntheses mixtures with chemical composition 1 SiO_2 : 0.05 Al_2O_3 : 0.18 Na_2O : 0.3R(OH) : 43.3H_2O after 4 and 7 days. Syntheses were performed under rotation (60 rpm) at 150 °C. The bottom spectrum in (a) and (b) are the liquid ^{1}H NMR and ^{13}C NMR of the N-benzyl-N,N-dimethylpropan-2-aminium iodide.

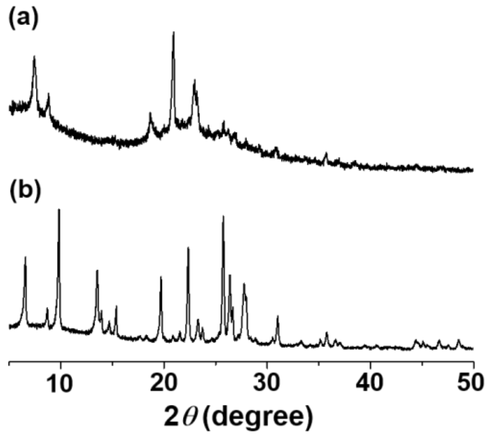

Figure S14. PXRD patterns (a) MOR + A and (b) ZSM-12 + A, where the product appearing first is the major phase and 'A' indicates amorphous phase. The solid materials were obtained from syntheses mixtures with chemical composition 1 SiO_2 : x Al_2O_3 : 0.18 Na_2O: 0.3 R(OH) : 43.3H_2O, where x = 0.025 and 0.05 for (a) and (b), respectively. Syntheses were performed under rotation (60 rpm) at 150 °C for 14 days.

MCM-58 type syntheses

The materials were synthesized from gels with the following composition:

$$SiO_2 : w\, Al_2O_3 : x\, K_2O : y\, R(OH) : z\, RI : 40\, H_2O$$

where R is the OSDA, and w, x, y, and z are varied between $0.05 < w < 0.005$, $0.00 < x < 0.29$, $0.00 < y < 0.56$, and $0.00 < z < 0.20$, respectively. The gels were prepared by mixing the aluminum sulfate octadecahydrate, KOH, OSDA source, and water for 1 hour. To this clear solution, colloidal silica was added and the mixture stirred for 4 hours. This mixture was then loaded into 23 mL Teflon-lined autoclaves and heated under rotation (60 rpm) at 175 °C for 7 days. The products were isolated using filtration, repeatedly washed with distilled water, and then dried at room temperature. Thereafter the Powder X-ray diffraction (PXRD) patterns were recorded on a PANalytical X'Pert diffractometer (Cu Kα radiation) with an X'Celerator detector.

Table S22. Results of synthesis using N-benzyl-N,N-dimethylpropan-2-aminium in MCM-58 type conditions.[a]

Si/Al	K_2O	RI	ROH	Product
10				A[b,c]
20	0.29	0.2	-	MOR + D
100				D
10				A[c]
20	-	-	0.56	A[c]
100				A[c]

[a]All syntheses were performed under rotation (60 rpm) at 175 °C for 7 days. [b] The product appearing first is the major phase. ‘A’ and ‘D’ indicate amorphous and dense phase, respectively. [c] Product obtained after 14 days

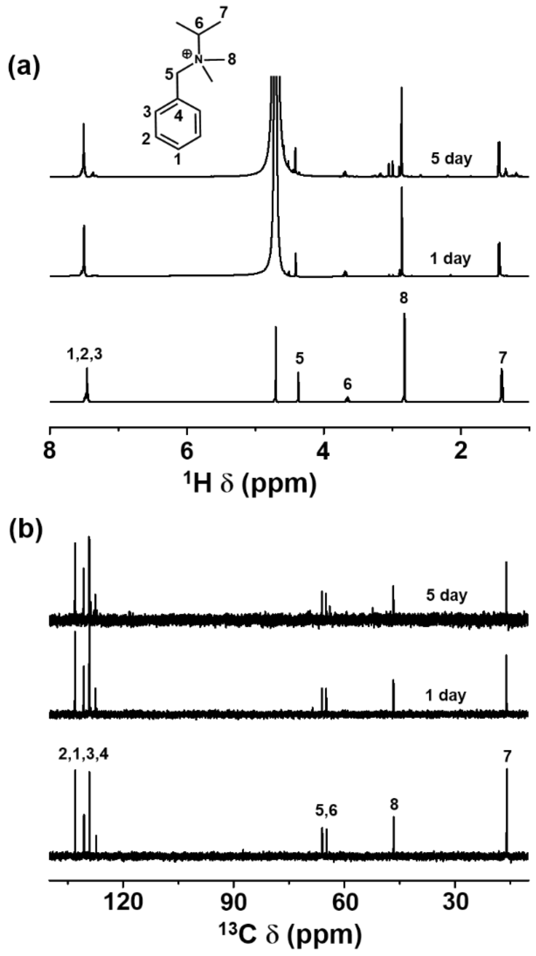

Figure S15. Liquid (a) ^{1}H NMR and (b) ^{13}C NMR of the N-benzyl-N,N-dimethylpropan-2-aminium cation obtained from mother liquors of syntheses mixtures with chemical composition 1 SiO_2 : 0.05 Al_2O_3 : 0.29 K_2O: 0.2 RI: 40 H_2O after 1 and 5 days. Syntheses were performed under rotation (60 rpm) at 175 °C. The bottom spectrum in (a) and (b) are the liquid ^{1}H NMR and ^{13}C NMR of the N-benzyl-N,N-dimethylpropan-2-aminium iodide.

References

(1) Altundal, O. F.; Leon, S.; Sastre, G. Different Zeolite Phases Obtained with the Same Organic Structure Directing Agent in the Presence and Absence of Aluminum: The Directing Role of Aluminum in the Synthesis of Zeolites. *J. Phys. Chem. C* **2023**, *127* (22), 10797-10805.

(2) Daeyaert, F.; Deem, M. W. Design of organic structure directing agents for polymorph A zeolite beta. *J. Mater. Chem. A* **2019**, *7* (16), 9854-9866.

(3) Schwalbe-Koda, D.; Gómez-Bombarelli, R. Benchmarking binding energy calculations for organic structure-directing agents in pure-silica zeolites. *J. Chem. Phys.* **2021**, *154* (17), 174109.

(4) Mayo, S. L.; Olafson, B. D.; Goddard, W. A. DREIDING: a generic force field for molecular simulations. *J. Phys. Chem.* **1990**, *94* (26), 8897-8909.

(5) Daeyaert, F.; Deem, M. W. In silico design of chiral dimers to direct the synthesis of a chiral zeolite. *Mol. Phys.* **2018**, *116* (21-22), 2836-2855.

(6) *Chemical Search*. https://chem-space.com/search (accessed 4/Nov/2025).

(7) Pophale, R.; Daeyaert, F.; Deem, M. W. Computational prediction of chemically synthesizable organic structure directing agents for zeolites. *J. Mater. Chem. A* **2013**, *1* (23), 6750-6760.

(8) Gálvez-Llompart, M.; Cantín, A.; Rey, F.; Sastre, G. Computational screening of structure directing agents for the synthesis of zeolites. A simplified model. *Z. fur Krist. - Cryst. Mater* **2019**, *234* (7-8), 451-460.

(9) Gale, J. D. GULP: A computer program for the symmetry-adapted simulation of solids. *J. Chem. Soc., Faraday Trans.* **1997**, *93* (4), 629-637.

(10) Lemishko, T.; Valencia, S.; Rey, F.; Jiménez-Ruiz, M.; Sastre, G. Inelastic neutron scattering study on the location of Brønsted acid sites in high silica LTA zeolite. *J. Phys. Chem. C* **2016**, *120* (43), 24904-24909.

(11) Oie, T.; Maggiora, G. M.; Christoffersen, R. E.; Duchamp, D. J. Development of a flexible intra-and intermolecular empirical potential function for large molecular systems. *Int. J. Quantum Chem.* **1981**, *20* (S8), 1-47.

(12) Bushuev, Y. G.; Sastre, G. Atomistic simulations of structural defects and water occluded in SSZ-74 zeolite. *J. Phys. Chem. C* **2009**, *113* (25), 10877-10886.

(13) Goldstein, H.; Poole, C.; Safko, J.; Addison, S. R. Classical mechanics. American Association of Physics Teachers: **2002**.

(14) Ewald, P. P. Die Berechnung optischer und elektrostatischer Gitterpotentiale. *Ann. Phys.* **1921**, *369* (3), 253-287.

(15) Rappé, A. K.; Casewit, C. J.; Colwell, K.; Goddard III, W. A.; Skiff, W. M. UFF, a full periodic table force field for molecular mechanics and molecular dynamics simulations. *J. Am. Chem. Soc.* **1992**, *114* (25), 10024-10035.

(16) Aitken, R. A.; Philp, E. F.; Riddell, F. G.; Smith, M. H. A synthetic and NMR conformational study of spiro cyclic quaternary ammonium salts. *ARKIVOC* **2002**, *2002* (3), 63-70.

(17) Corma, A.; Díaz-Cabañas, M. J.; Rey, F.; Nicolopoulus, S.; Boulahya, K. ITQ-15: The first ultralarge pore zeolite with a bi-directional pore system formed by intersecting 14-and 12-ring channels, and its catalytic implications. *Chem. Commun.* **2004**, (12), 1356-1357.

(18) Shvets, O. V.; Kasian, N.; Zukal, A.; Pinkas, J.; Čejka, J. The role of template structure and synergism between inorganic and organic structure directing agents in the synthesis of UTL zeolite. *Chem. Mater.* **2010**, *22* (11), 3482-3495.

(19) Elomari, S.; Zones, S. I. Method of Making Aluminum-Containing Zeolite with IFR Structure. US6821502B2, **2004**.

(20) Valyocsik, E. W. Synthesis of Porous Crystalline MCM-58. US5441721A, **1995**.
